# Global epidemiology of paralytic shellfish poisoning: a systematic search literature review

**DOI:** 10.1016/j.lanplh.2025.05.001

**Published:** 2025-08-13

**Authors:** Matthew O Gribble, Baylin J Bennett, Jahred M Liddie, William Borchert, Brigitte A Pfluger, Jackson S Segars, Jacob M Keast, Avneet Hans, Nidhi S Kikkeri, Caitlin Shin, Hugh B Roland, Sneha Hoysala, Jacob Kohlhoff, Barrak Alahmad, Shivaraj Nagalli, William Rushton, Henrik Enevoldsen, Megan Bell, Damiana Fortenberry, John R Harley, Andrea L C Schneider

**Affiliations:** Division of Occupational, Environmental, and Climate Medicine, Department of Medicine, University of California, San Francisco, CA, USA; Division of Occupational, Environmental, and Climate Medicine, Department of Medicine, University of California, San Francisco, CA, USA; Harvard T.H. Chan School of Public Health, Boston, MA, USA; Harvard T.H. Chan School of Public Health, Boston, MA, USA; Nutrition and Health Sciences, Laney Graduate School, Emory University, Atlanta, GA, USA; Department of Epidemiology, University of Alabama at Birmingham, Birmingham, AL, USA; University of Exeter Medical School, St. Luke’s Campus, Exeter, Devon, UK; Heersink School of Medicine, Birmingham, AL, USA; Heersink School of Medicine, Birmingham, AL, USA; California University of Science and Medicine, Colton, CA, USA; Department of Health Policy and Organization, University of Alabama at Birmingham, Birmingham, AL, USA; Rollins School of Public Health, Atlanta, GA, USA; Sitka Tribe of Alaska, Sitka, AK, USA; Harvard T.H. Chan School of Public Health, Boston, MA, USA; Shelby Baptist Medical Center, Alabaster, AL, USA; Heersink School of Medicine, Birmingham, AL, USA; Intergovernmental Oceanographic Commission Science and Communication Centre on Harmful Algae, IOC UNESCO, Copenhagen, Denmark; University of Alabama at Birmingham Libraries, Birmingham, AL, USA; University of Alabama at Birmingham Libraries, Birmingham, AL, USA; Alaska Coastal Rainforest Center, University of Alaska Southeast, Juneau, AK, USA; Department of Neurology, University of Pennsylvania Perelman School of Medicine, Philadelphia, PA, USA

## Abstract

We are in the midst of the UN Decade of Ocean Science for Sustainable Development (2021–30), which provides a timely opportunity for the epidemiological community to assess the global burden of thalassogenic diseases such as paralytic shellfish poisoning (PSP). In this epidemiological review, we used systematic search tools to summarise 152 peer-reviewed articles describing human PSP cases. Our analysis revealed that PSP cases have been reported from every inhabited continent; symptoms reported by patients might differ by continent; and exposure sources are not limited to the eponymic shellfish. Furthermore, most cases described lacked demographic details that could aid in a more comprehensive understanding of PSP epidemiology. Overall, this Review highlights PSP as a true global health concern; however, the overall poor quality of available data underscores the need for greater epidemiological attention as an understudied global health challenge.

## Introduction

We are in the midst of the UN Decade of Ocean Science for Sustainable Development (2021–30), a period designated by the UN as especially timely for advancing research efforts towards achieving a safe ocean where life and livelihoods are protected from ocean-related hazards.^1^ This decade presents a crucial opportunity for the public health research community to contribute to transdisciplinary dialogues on the intersection of oceans and human health.^2^ Within this framework, seafood is a crucial contributor to shaping nutrition and economies globally.^3^ However, chemical contaminants present in seafood pose considerable health risks that might counterbalance its potential nutritional benefits.^4^

Fisheries economists often focus on the economic consequences of harmful algal blooms (HABs), particularly those arising from commercial fishery closures intended to prevent human exposure to marine toxins.^5^ However, economic losses also stem from the persistent disease burden caused by HAB toxins despite preventive measures—eg, poisoning from toxins in non-monitored seafood, such as subsistence harvests.^6^ Since the incidence of HAB-related poisonings is increasingly linked to climate change, the associated health costs have implications for broader policy debates around the social cost of carbon that epidemiologists actively contribute to.^7^ Although regional reviews of paralytic shellfish poisoning (PSP) epidemiology exist—covering areas such as Alaska,^8^ Japan,^9^ Portugal,^10^ South Africa,^11^ Tasmania,^12^ and Venezuela^13^—a comprehensive synthesis of evidence of the global burden of PSP remains necessary.

The urgency for public health scientists to investigate the health effects of HABs has grown as climate change intensifies environmental uncertainties, and the number of reports of HAB events appears to be increasing, at least in the USA.^14,15^ PSP, caused by some HAB species such as *Alexandrium*,^16^ remains a crucial concern. However, substantial gaps persist in understanding how climate change influences the environmental risk factors for PSP, including the genetic, physiological, and ecological responses of harmful algae.^17^ Evidence suggests that climate change affects the growth conditions of marine harmful algae,^18^ including PSP-related taxa such as *Alexandrium*.^19^ Additionally, climate change and ocean acidification might alter the toxicokinetics of shellfish and the bioavailability of PSP toxins, thereby influencing human exposure.^20,21^ Given this evolving environmental risk landscape, a thorough review of the global epidemiology of PSP is essential.

Beyond the relevance of this Review to ocean-related health hazards, we also address a growing theme in neurology: the role of environmental risk factors in the global burden of neurological disorders.^22^ Although the global prevalence of common neurological diseases has risen over recent decades,^23,24^ temporal trends in neurological toxidromes such as PSP remain poorly understood. Some localised studies have examined PSP, such as an assessment of cases reported in British Columbia from 1941 to 2020.^25^ Yet, there is considerable value in updating and consolidating PSP data on a global scale to facilitate broader spatiotemporal trends.

The primary objective of this epidemiological literature review was to characterise the spatiotemporal distribution and population burden of PSP documented in peer-reviewed scientific publications. The secondary goal of this Review was to delineate PSP risk factors and clinical presentations.

## Methods

The review protocol was registered with PROSPERO as an epidemiological review (CRD42022376906). This Review was conducted in accordance with the PRISMA reporting guidelines.

### Search strategy and selection criteria

PubMed, Embase, Scopus, and Web of Science databases were searched on Aug 8, 2022, with updated searches on Feb 20, 2023, and March 26, 2024. The search strategy is detailed in the panel. The search strategy was designed to comprehensively identify epidemiological reports of PSP while enhancing the specificity of our search by excluding articles on toxic exposures unrelated to algal toxins that cause PSP (eg, mercury, arsenic, and pesticides) and non-human research.

This Review included all articles describing outcomes related to PSP, such as case reports or epidemiological literature, published in peer-reviewed journals, irrespective of language. Both primary research articles and review papers documenting specific instances of PSP in human populations were considered eligible. Articles focusing exclusively on non-human data—such as toxin concentrations in shellfish without reference to human cases or the presence of algal species without associated human poisoning—were excluded. Reports not published in peer-reviewed journals were also excluded.

### Selection process and data collection process

Each article was screened by two independent reviewers at both the title and abstract screening and full-text screening stages. Disagreements about article eligibility between reviewers were handled using post-hoc tie-breaking decisions after re-examination of the article, involving either consensus between the initial reviewers or the input of a third reviewer. Data extraction was likewise conducted independently by two reviewers per article, with any disagreements addressed using the post-hoc tie-breaking decision. Article screening, data abstraction, and tiebreaking decisions were conducted using Covidence Systematic Review Software (Veritas Health Innovation, Melbourne, Australia). Non-English articles were evaluated by study team members or collaborators fluent in the respective languages according to the same procedure as described for screening English articles. One Korean-language article identified as potentially relevant by a colleague was translated into English by LanguageLine Solutions (Monterey, California);^26^ all other non-English articles were evaluated in their original language.

### Data extraction and risk of bias assessment

The following data were extracted from each included article, as available and applicable: article title, surname of the first author, year of publication, journal name, affiliations of all authors (eg, institution and department), source of funding or sponsorship (eg, governmental, non-governmental, unsponsored, or not reported), article language, number of outbreaks reported, study design (eg, ecological/time-series/surveillance, case series, or other), definition of PSP cases, and demographic characteristics of affected individuals. PSP case definitions were categorised as follows: definite—symptoms attributed to PSP following seafood consumption with saxitoxin biomarker confirmation (in human urine or seafood consumed); probable—symptoms attributed to PSP following seafood consumption without biomarker confirmation; possible—symptoms described as potentially consistent with PSP after seafood consumption without biomarker confirmation; and not specified—typically observed in surveillance summaries reporting aggregate PSP case counts. Additionally, the following data were extracted for each outbreak: number of cases, start and end dates, symptom duration, number of hospitalised cases, number of cases requiring intensive care, duration of hospitalisation, number of deaths, time to death (per fatal case), geographical location, reported symptoms, time to earliest symptom onset (categorised as <30 min, 1–4 h, >4 to 12 h, ≥12 h, or not reported), and identified risk factors or exposures (eg, molluscs or shellfish, fish, algae or water, or unspecified). Further details included the specific exposure source, toxigenic algae involved, location of exposure (eg, home, vacation or tourist setting, or not reported), source of seafood (eg, personal fishing, clamming, or subsistence harvesting, purchased at a market or store, consumed in a restaurant or catered event, or not reported), and whether the study specified a target population to which the findings could be generalised. Data not included in articles were recorded as not reported.^27^

Given the heterogeneity of the included studies, an overall assessment of evidence quality was undertaken based on both reported data and a risk of bias evaluation. Although our PROSPERO-registered protocol (CRD42022376906), submitted as an “epidemiologic review” in PROSPERO), initially specified a risk of bias assessment adapted from the Risk Of Bias In Non-Randomised Studies of Interventions (ROBINS-I)^28^ and the Newcastle-Ottawa Scale,^29^ most of the included articles were case reports or case series. Therefore, we used a modified version of the Grading of Recommendations Assessment, Development and Evaluation (GRADE) framework to evaluate the risk of bias,^30^ which was assessed for each article based on the following indicators: author affiliations (institution and department), source of funding or sponsorship, study design, PSP case definition used, and whether the article defined a target population for generalisability. Two independent reviewers assessed each article against the criteria, with any discrepancies resolved through consensus or, if necessary, adjudication by a third reviewer.

### Effect measures and synthesis methods

Most articles included in this Review (eg, case reports and case series) were inadequate for quantitative estimates; therefore, a meta-analysis could not be performed. In lieu of effect measures, we summarised the findings of individual articles descriptively, including the use of contingency tables to illustrate relationships between variables such as the type of seafood consumed and symptoms reported.

## Results

### Literature review

The figure presents the flow diagram for article selection. The literature search yielded 7335 articles. After excluding 148 duplicate articles, titles and articles of 7188 articles were screened. 6837 articles were excluded, and 350 full-text articles were assessed for eligibility. Among the 350 full-text articles, one full-text article could not be retrieved,^31^ 180 were excluded for not reporting PSP outcomes in humans, and 17 were excluded for being non-peer-reviewed publications, resulting in 152 articles being finally included in the evaluation.^6,8–13,15,25,26,32–173^

### Results of individual articles

Key features of the individual articles are summarised in tables 1–3.

### Article characteristics

The characteristics of the 152 included articles are summarised in table 1, with additional details in the appendix (pp 1–6, 15–24). Most articles (127 [83⋅6%]) were written in English, and 91 (59⋅9%) articles were published before 2000. The median (25th–75th percentile) number of PSP outbreaks reported per article was one.^1–5^ PSP outbreaks were documented across all continents—Africa, the Americas, Asia, Europe, and Oceania. However, among the 135 articles that reported outbreaks confined to a single continent, 120 (88⋅9%) described outbreaks occurring in North America, Asia, or Europe. Demographic data (age, race or ethnicity, and sex) of PSP cases were infrequently reported: age was reported in 51 (37⋅8%) articles, race or ethnicity in 12 (8⋅9%), and sex in 52 (38⋅5%). Furthermore, PSP was reported to affect individuals across the lifespan—from infancy to more than 80 years of age—and occurred in both sexes.

Details of seafood exposure are presented by continent in table 2. Across all regions except Africa, the most common route of exposure was via the consumption of molluscs or other shellfish. In Africa, the specific type of seafood exposure was typically unspecified. The outbreak characteristics by continent and year (<2000 *vs* ^3^2000) are presented in table 3. Across all continents, 409 (77⋅0%) of 531 outbreaks were reported before 2000, during which the hospitalisation rates ranged from 2⋅3% (Europe) to 27⋅4% (South America). The percentage of cases requiring intensive care ranged from 0% (Africa and Europe) to 1⋅2% (Oceania). Case fatality rates ranged from 3⋅1% (Europe) to 12⋅3% (North America). Since 2000, hospitalisation rates have ranged from 0% (Africa and South America) to 60⋅0% (Oceania), whereas the proportion of cases requiring intensive care ranged from 0% (Africa, Asia, and Oceania) to 15⋅8% (Europe). The fatality percentage ranged from 0% (Africa, Europe, and Oceania) to 9⋅1% (North America). When symptom onset time was reported, most cases reported symptom onset time of less than 30 min in Africa (two [100%] of two), Asia (eight [57⋅1%] of 14), North America (20 [64⋅5] of 31), Oceania (two [50%] of four), and South America (four [100%] of four). In contrast, most cases (seven [63⋅6%] of 11) in Europe reported a symptom onset time of 1–4 h. The duration of reported symptoms ranged from 30 min to 45 days. The appendix (p 45) shows the percentage of clinical characteristics and symptoms reported by continent. Among articles that reported symptoms, perioral paraesthesia was the most common (ranging from ten [41⋅7%] of 24 in Europe to two [100%] of two in Africa and four [100%] of four in Oceania). Paralysis was the least reported symptom, ranging from two (8⋅3%) of 24 cases in Europe to one (50⋅0%) of two cases in Africa. Other reported symptoms across continents included paraesthesia in the arms and legs, ataxia or weakness, headache, dysarthria, respiratory distress, nausea or vomiting, dizziness, and dysphagia. No symptoms were reported in 61 (45⋅2%) of 135 articles (ranging from 0% in Africa and Oceania to 55⋅6% in South America).

### Findings of risk of bias and quality of evidence assessments

The appendix (pp 25–38) summarises the findings of risk of bias and quality of evidence assessments. Most articles (102 [67⋅1%] of 152) were authored by groups with at least one government affiliation, and 71 (46⋅7%) articles were authored by groups affiliated with one or more universities or academic institutions. Furthermore, 123 (80⋅9%) articles were published by author groups affiliated with the departments of medicine or public health. In contrast, only a few articles (51 [33⋅6%]) included author groups having at least one author affiliated with ecology or environmental science departments. Regarding funding, 40 (26⋅3%) articles were sponsored by government sources, six (4⋅0%) by non-government sources, three (2⋅0%) were unsponsored, and 110 (72⋅4%) did not report funding sources.

In terms of study design, the majority of articles (67 [44⋅1%] of 152) were either case series or case reports; 45 (29⋅6%) articles were ecological or time-series or surveillance articles; 38 (25⋅0%) were literature reviews that reported individual outbreak data; and two (1⋅3%) were classified as other (including case–control studies and chemical analyses linked to outbreak data). The studies evaluated in this Review included only one PSP community surveillance survey conducted on Kodiak Island in Alaska in 1994.^78^ The case definition used for PSP was categorised as definite in 51 (33⋅6%) articles, probable in 32 (21⋅1%), possible in five (3⋅3%), and not specified in 64 (42⋅1%). Only 19 (12⋅5%) articles identified a target population to which findings could be generalised.

Based on the available data and risk of bias assessment findings from the articles identified, which were largely case reports and outbreak summaries with only one population-based epidemiological article, the quality of evidence was poor.

## Discussion

The principal finding of this epidemiological review is that PSP represents a genuine global public health concern. PSP cases were reported across Africa, Asia, the Americas, Oceania, and Europe, with substantial consistencies in symptom profiles across these regions. Commonly reported symptoms included ataxia or weakness, nausea or vomiting, paraesthesia of the arms and legs, and perioral paraesthesia. Furthermore, dizziness or vertigo, dysarthria, paralysis, and respiratory distress have been reported across geographical regions. Although dysphagia was reported in more than three cases in North America (n=9), under-reporting of dysphagia is common.^176^ The presence of some symptoms in only one geographical area could reflect underlying case heterogeneity that is difficult to evaluate from the scarce epidemiological literature. Case subtype variation is a plausible basis for expecting PSP case subtype heterogeneity owing to the regional differences in the occurrence of PSP toxin-producing algae. For instance, although *Alexandrium* spp is the most well-known toxigenic algae,^177^ other genera such as *Gymnodinium catenatum* and *Pyrodinium bahamense* also produce PSP toxins.^177–179^

Therefore, the specific congener composition of toxins varies by algal taxa; however, transformations of toxins within marine organisms such as clams^180^ might expose consumers of different seafood organisms in the same contaminated ecosystem to different toxin mixtures. Therefore, comparative epidemiological surveys using standardised protocols in different geographical settings would be valuable to understand the possibility of case presentation differences and whether these differences vary according to environmental conditions and algal community composition.

A wide range of seafood species could be contaminated with PSP toxins. Although most cases are linked to the consumption of bivalve shellfish (eg, clams, mussels, cockles, and oysters), other vectors include sea snails, crabs, and, less commonly, finfish such as pufferfish and mackerel.^6,25,49,62,83,102,104,165,172^ Although PSP toxin exposure through marine biota other than shellfish has been described,^181–183^ alternative routes of exposure are more often characterised for marine wildlife such as whales and seabirds.^184^ Even though exposure to PSP toxins through sources other than bivalves is rare, monitoring programmes often focus only on bivalves.^185^ These findings suggest that greater attention should be paid to alternative vectors not yet completely incorporated into public health surveillance frameworks.

Several key limitations affected both the evidence base and our review process. First, the literature is dominated by case reports, case series, and outbreak summaries, which are insufficient for estimating the incidence of mild or moderate disease. Furthermore, case reports often highlight unusual or severe presentations, and typical cases can go undocumented in the peer-reviewed literature. Despite the availability of some quantitative data for broad comparisons (eg, historical numbers of cases in a region over a given period or the relative occurrence of outbreaks before *vs* after 2000), the data are not robust enough for estimating population-level incidence. The articles included in the analysis in this Review comprised case reports, case series, and outbreak investigations, with only one single population-based surveillance study from Kodiak Island, Alaska, conducted in 1994.^78^ This scarcity of systematic data makes drawing inferences about time trends in true population poisoning rates difficult rather than events sporadically published in peer-reviewed literature. Furthermore, several of the reports that we identified were redundant in describing the same outbreaks. For example, a collection of mussels from Spain was shipped to Switzerland, where they caused a mass outbreak of PSP among more than 200 people.^51,173^ This single outbreak event was described in contemporary articles published in multiple languages. The level of detail surrounding individual outbreaks was scarce in many reports, and many articles summarised historical outbreaks. Consequently, distinguishing between unique outbreaks across the epidemiological literature was deemed infeasible, representing an important limitation for burden estimation—particularly in the context of climate change, which is expected to influence the future risk of PSP.^14–18^ In addition to the published epidemiological literature being somewhat challenging to interpret, our review process itself had several limitations. Although we included results from relevant articles published in any language, budgetary barriers precluded paying for language translation services for every article. Therefore, we relied on colleagues for some of the decision making and data extraction for articles written in languages not spoken by our primary research team but for which we had at least two proficient-in-the-language contacts. These contributors are noted in the Acknowledgments. Finally, it is important to note that this Review focused solely on human cases. Toxins occurring in marine food webs are naturally a One Health topic, and there is also an important and growing literature on the veterinary and ecological implications of PSP-causing toxin exposures for marine wildlife that was beyond the scope of our review.^186,187^

This Review has implications for PSP disease prevention, public health tracking, and scientific opportunities in planetary health science. Clinicians and consumers should be aware that PSP can result from the consumption of a broader range of marine species beyond bivalves. Furthermore, this Review informs that public health practitioners could strengthen epidemiologic surveillance efforts by not limiting case definitions to require that people have consumed shellfish. Finally, in light of the need for a more systematic approach to epidemiological tracking of PSP and major ongoing efforts of the global ocean science community to protect the public from marine toxin hazards,^188^ including advancements in PSP monitoring^189^ and toxicology relevant to PSP,^190,191^ we want to echo other recent calls for public health scientists to engage in interdisciplinary collaborations around oceans and human health.^2^ Generating quantitative insights into the relative risks of PSP for consumers of various seafood types and evaluating the extent to which PSP case heterogeneity exists and has marine ecological determinants would require new interdisciplinary studies with epidemiologists working with marine biology input throughout the research process.

## Conclusions

The science of PSP epidemiology remains constrained by peer-reviewed literature that predominantly comprises outbreak reports rather than systematically collected data from larger populations at risk. Nevertheless, PSP cases have been documented on all continents, and some PSP cases occurred by exposure to toxins through seafood other than the eponymous shellfish, including crab, fish, sea snails, and tunicates. We recommend more interdisciplinary collaborations between public health practitioners and ocean scientists to better characterise the population burden, environmental risk factors, and phenotypic presentations of PSP.

## Supplementary Material

Supplementary appendix

## Figures and Tables

**Figure:**
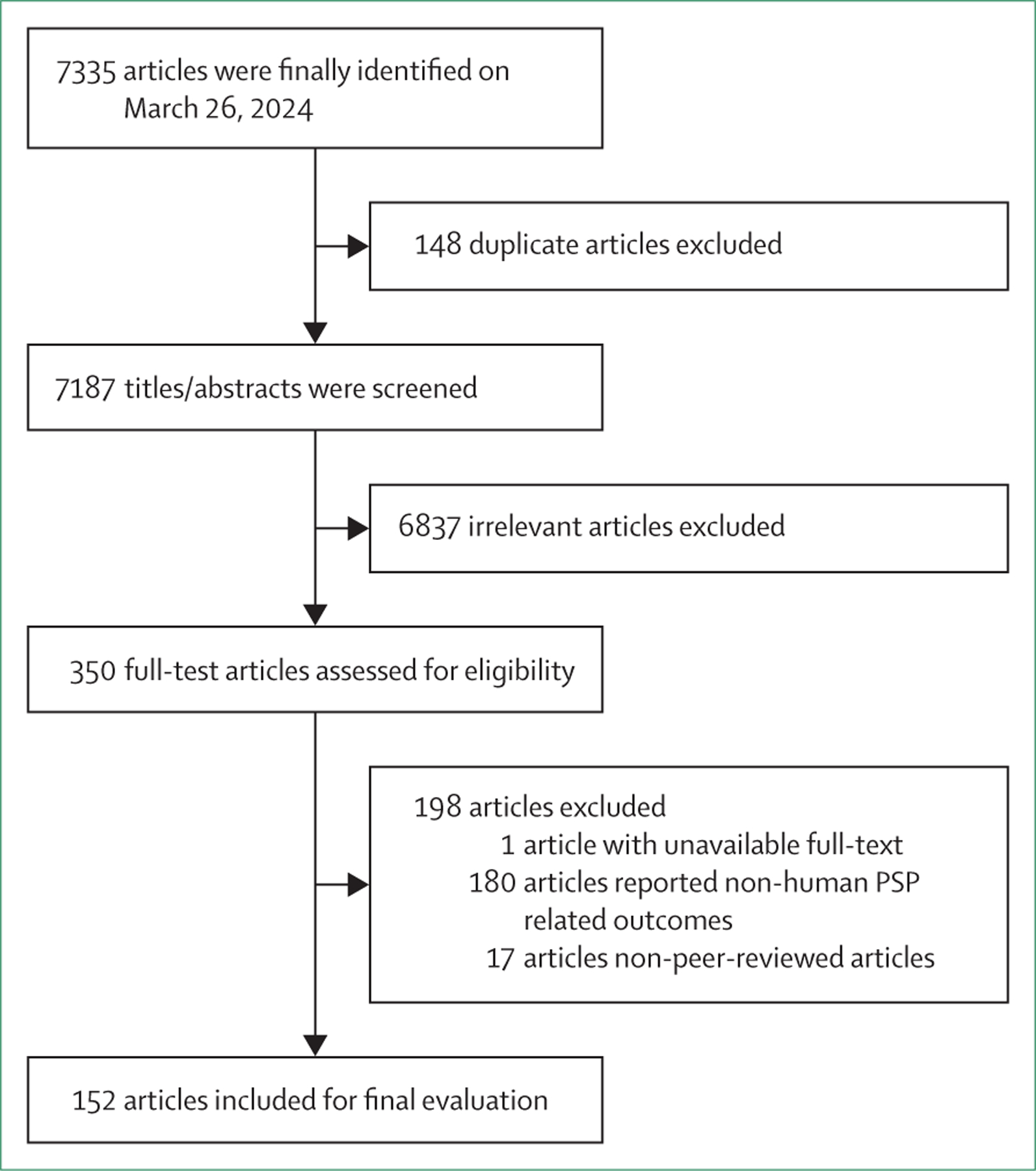
Flow diagram of study screening and selection for a review of global PSP outbreaks.

**Table 1:** Case demographics

	Age range										Race or ethnicity							Sex		
	0–9	10–19	20–29	30–39	40–49	50–59	60–69	70–79	≥80	Not reported	White	Black	Hispanic	Native or indigenous	Asian	Other*	Not reported	Female	Male	Not reported
**Africa**																				
McFarren et al (1960)^109^										X				>100						X
Popkiss et al (1979)^11^										X	17	0	0	0	0	0		7	10	
Batoréu et al (2005)^46^										X							X			X
Arnich and Thébault (2018)^35^	X	X	X	X	X	X	X										X	6	10	
Marks et al (2019)^105^										X							X			X
Vale (2020)^166^										X							X			X
**Asia**																				
Kawabata et al (1962)^96^										X							X			X
Roy (1977)^132^										X							X	5	4	
Imbert et al (1979)^91^										X							X			X
Gacutan et al (1985)^73^										X							X			X
Kan et al (1986)^94^	X	X		X													X	>3	>2	
Kim et al (1986)^26^			X	X	X	X											X	0	25	
Hashimoto and Noguchi (1989)^83^										X							X			X
MacLean (1989)^102^										X							X			X
Swaddiwudhipong et al (1989)^145^										X							X			X
Cheng et al (1991)^58^				X													X	2	3	
Viviani (1992)^169^										X							X			X
Negoro et al (1993)^122^				X	X	X		X	X								X	1	4	
Hartigan-Go and Bateman (1994)^82^	X	X	X	X	X	X	X	X									X			X
Todd (1994)^152^										X							X			X
Corrales and Maclean (1995)^65^										X							X			X
Hwang et al (1995)^90^										X							X			X
Akaeda et al (1998)^9^										X							X			X
Trevino (1998)^162^										X							X			X
Bankoff (1999)^42^										X							X			X
Morris (1999)^118^										X							X			X
Murakami and Noguchi (2000)^121^										X							X			X
Azanza and Taylor (2001)^38^										X							X			X
Bajarias et al (2002)^40^										X							X			X
Holmes and Teo (2002)^87^										X							X			X
Batoréu et al (2005)^46^										X							X			X
Azanza (2006)^37^										X							X			X
Chung et al (2006)^60^		X	X	X	X	X	X	X	X								X	32	26	
Jen et al (2008)^93^						X											X			X
James et al (2010)^92^										X							X			X
Toda et al (2012)^150^										X							X			X
Ching et al (2015)^59^	X	X	X	X	X	X	X										X	13	18	
Suleiman et al (2017)^143^	X	X	X	X	X	X	X										X	34	24	
Arnich and Thébault (2018)^35^	X	X	X	X	X	X	X										X			X
Azzeri et al (2020)^39^										X							X			X
Velayudhan et al (2021)^168^	X	X	X	X	X	X	X	X									X	116	103	
Chen et al (2023)^57^	X								X						55			26	29	
Yu et al (2023)^171^										X							X			X
Zheng et al (2023)^172^					X	X	X										X	2	1	
McFarren et al (1960)^109^										X				>100						X
McCollum et a (1968)^108^										X							X			X
Ayres et al (1975)^36^										X							X			X
Blanc et al (1977)^50^										X							X			X
Zwahlen et al (1977)^173^										X							X			X
Blanc et al (1978)^51^			X														X			X
Caroli et al (1978)^55^	X	X	X	X	X	X	X										X	12	14	
Imbert et al (1979)^91^										X							X			X
Gulbrandsen et al (1981)^81^			X	X							4	0	0	0	0	0		2	2	
Tangen (1983)^146^										X							X			X
Hasselgård and Hjelle (1984)^84^	X	X	X	X	X	X											X	6	4	
Langeland et al (1984)^98^	X	X	X	X		X											X	4	4	
Sanders (1987)^135^										X							X			X
Mills and Passmore (1988)^114^										X							X			X
No authors listed (1988)^124^			X							X							X			X
The PHLS Communicable Disease Surveillance Centre (1990)^149^										X							X			X
Scoging (1991)^137^										X							X			X
Viviani (1992)^169^										X							X			X
Ledoux and Frémy (1994)^99^										X							X			X
Martin et al (1996)^106^										X							X			X
de Carvalho et al (1998)^10^				X	X	X	X	X	X								X	6	3	
Scoging (1998)^138^										X							X			X
Krys and Frémy (2002)^97^										X							X			X
Batoréu et al (2005)^46^										X							X			X
Mira Gutiérrez (2005)^115^										X							X			X
Rapala et al (2005)^127^	X	X															X			X
James et al (2010)^92^										X							X			X
Hinder et al (2011)^86^										X							X			X
Arnich and Thébault (2018)^35^	X	X	X	X	X	X	X										X	5	5	
Carvalho et al (2019)^56^							X										X	1	1	
Vale (2020)^166^										X							X	1	2	
Karlson et al (2021)^95^										X							X			X
Sinno-Tellier et al (2022)^140^		X	X	X	X	X	X										X	7	8	
Rodríguez et al (2024)^131^										X							X			X
**North America**																				
Scobey (1947)^136^		X															X			X
Meyers and Hilliard (1955)^113^										X							X	0	1	
Tennant et al (1955)^148^		X	X	X			X										X	5	2	
Bond and Medcof (1958)^52^	X		X	X	X	X	X										X	15	18	
McFarren et al (1960)^109^										X				>100				0	>100	
Meinke and Quinn (1973)^112^						X											X	0	1	
Fortuine (1975)^71^										X							X			X
Cladouhos (1977)^61^							X										X	1	1	
Craun (1977)^67^										X							X			X
Hughes et al (1977)^88^										X							X			X
Morse (1977)^119^										X							X			X
Todd (1977)^154^										X							X			X
Acres and Gray (1978)^32^						X											X	0	2	
Imbert et al (1979)^91^										X							X			X
Bryan (1980)^53^										X							X			X
Grimard and Lalonde (1981)^80^					X		X										X	1	1	
Todd (1982)^155^										X							X			X
de la Garza Aguilar (1983)^68^	X	X	X	X	X												X	7	12	
No authors listed (1983)^123^										X							X			X
Conte (1984)^64^										X							X			X
Todd (1985)^156^										X							X			X
Todd (1985)^157^										X							X			X
Mee et al (1986)^111^	X	X		X													X	5	13	
Sanders (1987)^135^										X							X			X
Todd (1987)^158^										X							X			X
Mills and Passmore (1988)^114^										X							X			X
No authors listed (1988)^124^			X							X							X			X
Todd (1988)^159^										X							X			X
Todd (1989)^160^										X							X			X
Long et al (1990)^101^						X											X	1	0	
Mata et al (1990)^107^					X		X										X			X
No authors listed (1990)^125^										X							X			X
Rodrigue et al (1990)^130^	X	X															X	99	88	
Ahmed (1991)^33^										X							X			X
No authors listed (1991)^126^			X	X	X												X	0	6	
Saldate Castañeda et al (1991)^134^										X							X			X
Saldate Castañeda et al (1991)^134^										X							X			X
United States Centers for Disease Control and Prevention (1991)^164^			X	X	X						0	0	0	1	0	0		0	6	
Ahmed (1992)^34^										X							X			X
Viviani (1992)^169^										X							X			
Moss (1993)^120^										X				135				0	135	
Saavedra-Deigado and Metcalfe (1993)^133^										X							X			X
Todd et al (1993)^151^				X		X											X	0	2	
Cortés-Altamirano et al (1995)^66^										X							X			X
Gessner and Middaugh (1995)^77^	X	X	X	X	X	X	X	X			37	0	0	33	12	0		44	66	
Bean et al (1996)^48^										X							X			X
Gessner and Schloss (1996)^78^										X	0	0	0	12	0	1*	X	8	5	
Bean et al (1997)^47^										X							X			X
Gessner et al (1997)^8^			X	X	X	X	X				0	0	2	2	7	0		6	5	
Gessner et al (1997)^76^			X										1					0	1	
Todd (1997)^153^										X							X			X
Sierra-Beltrán et al (1998)^139^										X							X			X
Trevino (1998)^162^										X							X			X
Bankoff (1999)^42^										X							X			X
Morris (1999)^118^										X							X			X
United States Centers for Disease Control and Prevention (2002)^165^			X	X		X	X			X							X	1	4	
United States Centers for Disease Control and Prevention (2002)^165^										X							X			X
Balmer-Hanchey et al (2003)^41^										X							X			X
Barbier and Diaz (2006)^43^										X							X			X
Batoréu et al (2005)^46^										X							X			X
Sobel and Painter (2005)^142^										X							X			X
Fortuine (2007)^72^										X	0	0	0	1+	0	0		0	1+	
Wang (2008)^170^										X							X			X
Barraza (2009)^44^	X	X															X	4	0	
James et al (2010)^92^										X							X			X
Bienfang et al (2011)^49^										X							X			X
McLaughlin et al (2011)^110^										X							X			X
Gould et al (2013)^79^										X							X			X
DeGrasse et al (2014)^69^										X							X			X
Hurley et al (2014)^89^		X			X	X	X										X	3	4	
Trainer et al (2014)^161^										X							X			X
Callejas et al (2015)^54^										X							X			X
Clemence and Guerrant (2015)^62^										X							X			X
Knaack et al (2016)^6^			X	X	X	X	X										X			X
Arnich and Thébault (2018)^35^	X	X	X	X	X	X	X										X	30	48	
Coleman et al (2018)^63^										X							X	1	0	
Anderson et al (2021)^15^										X							X			X
McIntyre et al (2021)^25^	X	X	X	X	X	X	X	X	X		0	0	0	27	0	84	X	48	63	
Sunesen et al (2021)^144^										X							X			X
Temple and Hughes (2022)^147^							X										X	1	0	
Reséndiz-Colorado et al (2023)^128^										X							X			X
**Oceania**																				
McFarren et al (1960)^109^										X				>100						X
Rhodes et al (1975)^129^	X	X	X														X	15	8	
Eason and Harding (1987)^70^			X		X												X	2	1	
MacLean (1989)^102^										X							X			X
Bankoff (1999)^42^										X							X			X
Lehane (2001)^100^										X							X			X
Turnbull et al (2013)^163^										X							X			X
Arnich and Thébault (2018)^35^	X	X	X	X	X	X	X										X	0	1	
Edwards et al (2018)^12^						X	X										X	1	3	
**South America**																				
Vecchio et al (1986)^167^	X	X		X			X										X			X
Montebruno (1993)^116^									X								X	6	144	
Montebruno (1993)^117^	X		X	X			X										X	1	9	
García et al (2004)^74^										X							X	0	2	
La Barbera-Sánchez et al (2004)^13^										X							X			X
García et al (2005)^75^						X											X			X
Hernández et al (2005)^85^										X							X			X
James et al (2010)^92^										X							X			X
Arnich and Thébault (2018)^35^	X X	X	X	X	X	X											X	0	6	
Barría et al (2022)^45^										X							X			X
Mafra et al (2023)^103^										X							X			X
**Global**																				
Lehane (2001)^100^										X							X			X
Sinno-Tellier et al (2023)^141^										X							X			X

* Other represents the studies in which only one race or ethnicity was reported while that of several participants was not defined (eg, Gessner and Schloss (1996)^78^). Ages and races or ethnicities were not stratified by continent; therefore, if they were described in case descriptions, we indicated them with an “X” in the table. However, as sex was stratified, we listed the count for each sex when identified. We report our results with fidelity to our data extraction form (appendix pp 39–44).

**Table 2:** Seafood exposure details

	Outbreaks (*n*)	Risk factors or exposures (%)					Specific food or specific exposure	Toxigenic algae responsible	Shellfish origin (%)				
		Molluscs or shellfish	Fish	Algae or water	Multiple exposures	Unspecified			Personal	Bought at market or store	Consumed in restaurant or catered party	Multiple exposures	Not reported
Africa	3	100%	0	0	0	0	Blue mussels (*Mytilus edulis* L), Donax clams (Unspecified species)	*Gymnodinium catenatum*	0	0	33%	0	67%
Asia	76	66%	0	0	11%	24%	Asian moon scallops (*Amusium pleuronectes*), Asiatic hard clam (*Meretrix meretrix*), Black olive (*Oliva vidua fulminans*), Blue mussels (*Mytilus edulis* L), Comb pen shell (*Atrina pectinata*), Flag pen shell (*Atrina vexillum*), Fanshell clam (*Pinna muricata*), Flat oyster (*Ostrea edulis*), Giant ezo (*Mizuhopecten yessoensis*), Green mussels (*Mytilus smaragdinus* and *Perna viridis*), Korean mud snail (*Bullacta exarata*), Purple clams (*Nuttallia obscurata*), Queen scallop (*Aequipecten opercularis)*, Sea pineapple (*Halocynthia roretzi*), Short-necked clams (*Paratapes undulatus*)	*Alexandrium tamarense (A tamarensis),* *Gymnodinium catenatum, Pyrodinium bahamense* var *compressum*	13%	12%	0	9%	66%
**Europe**	76	54%	0	0	30%	16%	Blue mussels (*Mytilus edulis* L), Butter clams (Saxidomus giganteus*), Dog cockles (*Glycymeris glycymeris*), Donax clams (Unspecified species), Pacific littleneck clams (Protothaca staminea*), Northumbrian mussel (Unspecified species), Whelks (Unspecified species)	*Alexandrium catenella (Gonyaulax excavata),* *Alexandrium tamarense (A tamarensis),* *Gymnodinium catenatum,* *Prorocentrum cordatum (P minimum)*	5%	4%	1%	1%	88%
**North America**	337	53%	1%	0	25%	21%	Black clams (*Villorita cyprinoides*), Blue mussels (*Mytilus edulis*), Butter clams (Saxidomus giganteus*), California mussels (*Mytilus californianus*), Crab (Unspecified species), Concha negra clams (*Anadara tuberculosa*), Cow oyster (Spondylus calcifer*), Dog winkle (*Nucella lapillus*), Heart cockles (*Clinocardium nuttalli*), Mackerel (Unspecified species), Pacific littleneck clam (Protothaca staminea*), Pufferfish (Unspecified species), Razor clams (*Siliqua patula* and *Tagelus* sp), Rock oysters (*Crassostrea iridescens and Striostrea prismatica*), Scad (Unspecified species), Soft shell clams (*Mya arenaria*), Sea snail (Unspecified species)	*Alexandrium catenella,* *Alexandrium monilatum,* *Lingulodinium poleydra (Gonyaulax poliedrum*†), *Alexandrium tamarense (A tamarensis),* *Donax kindermanni (Amphichaena kindermani)*, *Ceratium virgatum (C rubrum),* *Dinophysis acuta,* *Gymnodinium catenatum,* *Mesodinium rubrum,* *Plicopurpura columellaris, Prorocentrum cordatum (P minimum),* *Pyrodinium bahamense* (including *compressum* variant)	11%	0	0	29%	59%
**Oceania**	5	100%	0	0	0	0	Mediterranean mussel (*Mytilus galloprovincialis*), Rock oysters (*Crassostrea gigas*)	*Alexandrium tamarense (A tamarensis)*	100%	0	0	0	0
**South** **America**	35	17%	0	0	0	83%	Chilean mussels (*Mytilus platensis*), Ribbed mussels (*Aulacomya ater*)	*Alexandrium catenella,* *Gonyaulax polygramma,* *Alexandrium tamarense (A tamarensis),* *Tripos furca (Ceratium furca),* *Chaetoceros neogracile (C gracilis)*^174^ *Margalefidinium polykrikoides (Cochlodinium polikrikoides),* *Cyclotella* sp, *Dinophysis* cf *acuminata,* *Dinophysis acuta,* *Mesodinium rubrum,* *Navicula* sp, *Neoporphyra seriata,* *Nuclearia delicatula,* *Pyrodinium bahamense*	6%	0	0	0	94%
**Global**	≥200	0	0	0		100%	Unspecified species	Unspecified species	0	0	0		100%

Percentages are based on continent-specific outbreak totals. The Personal category in Shellfish origin column included personal fishing, clamming, and subsistence harvesting. Articles reporting outbreaks from multiple continents (n=16) were excluded from this table because these categories were not stratified by continent, but articles reporting multiple outbreaks from a single continent were included. Sinno-Tellier and colleagues^141^ were excluded due to indiscriminate geography, and literature reviews were excluded due to no continent stratification for these data. Some algal species mentioned in articles are synonyms or no longer accepted species names; in these cases, we use the currently accepted species name followed by the name mentioned in the article in parentheses. Unless otherwise noted, taxonomic identifications were from a study by Guiry and Guiry.^175^

* The species names reported in the original sources are no longer valid but are reported here for fidelity.

† Authors listed *Gonyaulax poliedrum*; they were probably referring to *Gonyaulax polyedra*, which is now known as *Lingulodinium polyedra*.

**Table 3:** Outbreak severity

	Number of cases	% hospitalised (*n*)	% required intensive care (*n*)	% died (*n*)	Earliest onset time (%)					Minimum and maximum reported duration of symptoms
					<30 min	1–4 h	>4–12 h	>12 h	Not reported	
Africa before 2000	≥207	6⋅8% (14)	0	5⋅8% (12)	100%	0	0	0	0	36–48 h
Africa during or after 2000	23	0	0	0						36–48 h
Asia before 2000	≥7854	5⋅6% (443)	1	5⋅0% (396)	31%	19%	0	0	50%	2 h to 9 days
Asia during or after 2000	≥574	16⋅0% (92)	0	5⋅7% (33)						1 h to 9⋅5 days
Europe before 2000	≥4447	2⋅3% (101)	0	3⋅1% (140)	17%	30%	0	4%	48%	9 h to 3 months
Europe during or after 2000	≥19	57⋅9% (11)	15⋅8% (3)	0						Not reported
North America before 2000	≥10 187	2⋅7% (279)	0⋅2% (16)	12⋅3% (1258)	32%	15%	0	0	50%	30 min to 18 days
North America during or after 2000	≥438	4⋅6% (20)	1⋅6% (7)	9⋅1% (40)						2 h to 45 days
Oceania before 2000	≥81	23⋅5% (19)	1⋅2% (1)	11⋅1% (9)	75%	25%	0	0	0	1 week
Oceania during or after 2000	≥5	60⋅0% (3)	0	0						31 h
South America before 2000	≥1375	27⋅4% (377)	0⋅4% (5)	7⋅4% (102)	50%	0	0	0	50%	Not reported
South America during or after 2000	≥204	0	2⋅0% (4)	7⋅8% (16)						3–18 h

Percentages are based on the continent-specific and time-specific case totals. When cases were reported as ≥X, X was used to produce conservative percentages. The percentage of earliest onset time was calculated using total outbreaks rather than total cases. One study was excluded due to indiscriminate geography,^141^ nine studies were entirely excluded due to indiscriminate temporality,^35,38,69,89,100,101,127,144,147^ six studies were excluded for having outbreak periods that spanned our time stratification (eg, the outbreak started in 1999 and ended in 2008),^25,37,40,79,85,128^ and one study had instances of both.^142^

## *Panel:* Search queries used in each database on Aug 8, 2022, with updated searches on Feb 20, 2023, and March 26, 2024 (restricted to document-type article, early access).

### PubMed

- ((((“paralytic shell fish” OR “paralytic shellfish” OR “PSP” OR saxitoxin* OR “Shellfish Poisoning”[MeSH] OR (Neurotoxicity Syndromes[MeSH] OR “Neurotoxins”[MeSH] OR “Poisons”[MeSH] OR “toxin” OR “dinoflagellate*” OR “phytoplankton” OR “harmful algal bloom*” OR “Harmful Algal Bloom”[MeSH] OR “paresthesia” OR “dysesthesia” OR “Paresthesia”[MeSH] OR ((“paralysis” OR “Paralysis”[MesH] OR “respiratory failure” OR “Respiratory Insufficiency”[MeSH] OR “mechanical ventilation” OR “Respiration, Artificial”[MeSH] OR “orotracheal intubation” OR “Intubation, Intratracheal”[MeSH]) NOT (“Vocal Cord Paralysis”[MeSH] OR “vocal fold*” OR “airway stenosis” OR sleep-related))) AND ((“shellfish” OR “seafood*” OR “sea food*” OR ((“marine” OR “coastal” OR “traditional” OR “subsistence”) AND (“nutrition” OR “diet” OR food* OR “harvest”)) OR “eating” OR “ate the” OR “mollusc” OR “Mollusca”[MeSH] OR “Saxidomus” OR “clam” OR “mussel” OR “oyster” OR “geoduck” OR “lifeway*”) NOT (“arsenic” OR “mercury” OR “Hg” OR “methylmercury” OR “MeHg” OR “methyl mercury” OR “hydrargyrum” OR “pesticide”))))

### AND

- (epidem* OR “pandemic” OR “Case Series” OR “public health” OR “Public Health”[MeSH] OR “Environment and Public Health”[MeSH] OR “clinical toxicology” OR “toxinology” OR “prevalence” OR “Prevalence”[MeSH] OR “morbidity” OR “Morbidity”[MeSH] OR “incidence” OR “Incidence”[MeSH] OR “mortality” OR “Mortality”[MeSH] OR “population” OR “surveillance” OR “Population Surveillance”[MeSH] OR “Population Health”[MeSH] OR “Poison Control Centers”[MeSH] OR “Poison Information*” OR “Poison Center*” OR “Emergency Room*” OR “Emergency Service, Hospital” [mesh] OR “travel medicine” OR “Travel Medicine”[MeSH] OR “Population Health Management”[MeSH] OR “Vulnerable Populations”[MeSH] OR “death” OR “died” OR “diagnosis” OR “diagnosed” OR “reported” OR “vital statistics” OR “Vital Statistics”[MeSH])) NOT (“animals”[MeSH Terms] NOT “humans”[MeSH Terms])) NOT (“exposure assessment” OR “risk assessment” OR “Risk Assessment”[MeSH])

### Embase

- ((‘paralytic shellfish poisoning’/exp OR ‘paralytic shellfish’ OR “paralytic shell fish”) AND ‘article’/it) NOT (‘animals’/exp NOT ‘humans’/exp)

### Scopus

- ((TITLE-ABS-KEY (“paralytic shell fish” OR “paralytic shellfish” OR psp OR saxitoxin* OR toxin OR dinoflagellate* OR phytoplankton OR “harmful algal bloom” OR paresthesia OR dysesthesia OR “paralysis” OR “respiratory failure” OR “mechanical ventilation” OR “orotracheal intubation”)) AND (TITLE-ABS-KEY (shellfish OR “seafood” OR “sea food” OR “eating” OR “ate the” OR “mollusc” OR “Saxidomus” OR “clam” OR “mussel” OR “oyster” OR “geoduck” OR “lifeway”) AND (TITLE-ABS-KEY (epidem* OR “pandemic” OR “Case Series” OR “public health” OR “clinical toxicology” OR toxinology OR preva-lence OR morbidity OR incidence OR mortality OR population OR surveillance OR “Poi-son Information” OR “Poison Center” OR “Emergency Room” OR “travel medicine” OR “death” OR “died” OR diagnosis OR diagnosed OR reported OR “vital statistics”))) AND NOT (ALL (“exposure assessment” OR “risk assessment” OR “vocal fold” OR “airway ste-nosis” OR “sleep related” OR arsenic OR mercury OR hg OR methylmercury OR mehg OR “methyl mercury” OR hydrargyrum OR pesticide)) AND (LIMIT-TO (DOCTYPE, “ar”))

### Web of Science

- ALL=(“paralytic shellfish” OR “paralytic shell fish”) NOT ALL=(“exposure assessment” OR “risk assessment” OR “vocal fold” OR “airway stenosis” OR “sleep related” OR arsenic OR mercury OR Hg OR methylmercury OR MeHg OR “methyl mercury” OR hydrargyrum OR pesticide)

## Data sharing

The dataset generated in this review has been registered with Harvard Dataverse, a public data repository, at https://doi.org/10.7910/DVN/ARDWAU.
