# Supplementary material for: Global epidemiology of paralytic shellfish poisoning: a systematic search literature review
Source: Lancet Planet Health. Author manuscript; Available in PMC 2025 Sep 27. (PMC12476144; doi:10.1016/j.lanplh.2025.05.001)

THE LANCET
Planetary Health

Supplementary appendix

This appendix formed part of the original submission and has been peer reviewed.
We post it as supplied by the authors.

Supplement to: Gribble MO, Bennett BJ, Liddie JM, et al. Global epidemiology of paralytic shellfish poisoning: a systematic search literature review. *Lancet Planet Health* 2025.
https://doi.org/10.1016/j.lanplh.2025.05.001

SUPPLEMENTAL MATERIALS*

TABLE S1 ………………………………………………………………………… 1 – 6

REFERENCES ……………………………………………………………………… 7 – 14

TABLE S2 ……………………………………………………………………….. 15 – 24

TABLE S3 ………………………………………………………………………... 25 – 38

TEXT S1 ………………………………………………………………………….. 39 – 44

FIGURE S1 ……………………………………………………………………… 45

*__NOTE__: The raw data we collated for the purposes of this manuscript are publicly available on Harvard Dataverse at https://doi.org/10.7910/DVN/ARDWAU.

TABLE S1. Peer-Reviewed Articles Used in the Epidemiologic Review listing author and year by continent. References are sorted chronologically then alphabetically. "Global" refers to an article that did not differentiate which continent the discussed outbreak(s) occurred on.

First Author	Year	Africa	Asia	Europe	North America	Oceania	South America	Global
Scobey	1947				X			
Meyers	1955				X			
Tennant	1955				X			
Bond	1958				X			
McFarren	1960	X		X	X	X		
Kawabata	1962		X					
McCollum	1968			X				
Meinke III	1973				X			
Ayres	1975			X				
Fortuine	1975				X			
Rhodes	1975					X		
Blanc	1977			X				
Cladouhos	1977				X			
Craun	1977				X			
Hughes	1977				X			
Morse	1977				X			
Roy	1977		X					
Todd	1977				X			
Zwahlen	1977			X				
Acres	1978				X			
Blanc	1978			X				
Caroli	1978			X				
Imbert	1979		X	X	X			
Popkiss	1979	X						
Bryan	1980				X			
Grimard	1981				X			
Gulbrandsen	1981			X				
Todd	1982				X			

de la Garza Aguilar	1983				X			
None Listed	1983				X			
Tangen	1983			X				
Conte	1984				X			
Hasselgård	1984			X				
Langeland	1984			X				
Gacutan	1985		X					
Todd	1985				X			
Todd	1985				X			
Kan	1986		X					
Kim	1986		X					
Mee	1986				X			
Vecchio	1986						X	
Eason	1987					X		
Sanders	1987			X	X			
Todd	1987				X			
Mills	1988			X	X			
None Listed	1988			X	X			
Todd	1988				X			
Hashimoto	1989		X					
MacLean	1989		X			X		
Swaddiwudhipong	1989		X					
Todd	1989				X			
Long	1990				X			
Mata	1990				X			
None Listed	1990				X			
Rodrigue	1990				X			
The PHLS Communicable Disease Surveillance Centre	1990			X				
Ahmed	1991				X			

Cheng	1991		X					
None Listed	1991				X			
Saldate Castañeda	1991				X			
Scoging	1991			X				
United States Centers for Disease Control and Prevention	1991				X			
Ahmed	1992				X			
Viviani	1992		X	X	X			
Montebruno	1993						X	
Montebruno	1993						X	
Moss	1993				X			
Negoro	1993		X					
Saavedra-Deigado	1993				X			
Todd	1993				X			
Hartigan-Go	1994		X					
Ledoux	1994			X				
Todd	1994		X					
Corrales	1995		X					
Cortés-Altamirano	1995				X			
Gessner	1995				X			
Hwang	1995		X					
Bean	1996				X			
Gessner	1996				X			
Martin	1996			X				
Bean	1997				X			
Gessner	1997				X			
Gessner	1997				X			
Todd	1997				X			
Akaeda	1998		X					
de Carvalho	1998			X				

Scoging	1998			X				
Sierra-Beltrán	1998				X			
Trevino	1998		X		X			
Bankoff	1999		X		X	X		
Morris Jr	1999		X		X			
Murakami	2000		X					
Azanza	2001		X					
Lehane	2001					X		X
Bajarias	2002		X					
Holmes	2002		X					
Krys	2002			X				
Marcus	2002				X			
United States Centers for Disease Control and Prevention	2002				X			
Balmer-Hanchey	2003				X			
Barbier	2003				X			
García	2004						X	
La Barbera-Sánchez	2004						X	
Batoréu	2005	X	X	X	X		X	
García	2005						X	
Hernández	2005						X	
Mira Gutiérrez	2005			X				
Rapala	2005			X				
Sobel	2005				X			
Azanza	2006		X					
Chung	2006		X					
Fortuine	2007				X			
Jen	2008		X					
Wang	2008				X			

Barraza	2009				X			
James	2010		X	X	X		X	
Bienfang	2011				X			
Hinder	2011			X				
McLaughlin	2011				X			
Toda	2012		X					
Gould	2013				X			
Turnbull	2013					X		
DeGrasse	2014				X			
Hurley	2014				X			
Trainer	2014				X			
Callejas	2015				X			
Ching	2015		X					
Clemence	2015				X			
Knaack	2016				X			
Suleiman	2017		X					
Arnich	2018	X	X	X	X	X	X	
Coleman	2018				X			
Edwards	2018					X		
Carvalho	2019			X				
Marks	2019	X						
Azzeri	2020		X					
Vale	2020	X		X				
Anderson	2021				X			
Karlson	2021			X				
McIntyre	2021				X			
Sunesen	2021				X		X	
Velayudhan	2021		X					
Barría	2022						X	
Sinno-Tellier	2022			X				
Temple	2022				X			
Chen	2023		X					

Mafra Jr.	2023						X	
Reséndiz-Colorado	2023				X			
Sinno-Tellier	2023							X
Yu	2023		X					
Zheng	2023		X					
Rodríguez	2024			X				

REFERENCES

1. Scobey RR. Paralytic shell-fish disease and poliomyelitis. *Arch Pediatr (N Y)* 1947; **64**(7): 350-63.
2. Meyers HF, Hilliard DK. Shellfish poisoning episode in False Pass, Alaska. *Public Health Rep (1896)* 1955; **70**(4): 419-20.
3. Tennant AD, Naubert J, Corbeil HE. An outbreak of paralytic shellfish poisoning. *Can Med Assoc J* 1955; **72**(6): 436-9.
4. Bond RM, Medcof JC. Epidemic shellfish poisoning in New Brunswick, 1957. *Can Med Assoc J* 1958; **79**(1): 19-24.
5. McFarren EF, Schafer ML, Campbell JE, Lewis KH, Jensen ET, Schantz EJ. Public health significance of paralytic shellfish poison. *Adv Food Res* 1960; **10**: 135-79.
6. Kawabata T, Kubota Y, Yoshida T. Paralytic Shellfish Poison–I a Note On the Shellfish Poisoning Occurred in Ofunato City, Iwate Prefecture in May, 1961. *Nippon Suisan Gakkai Shi* 1962; **28**(3): 344.
7. McCollum JP, Pearson RC, Ingham HR, Wood PC, Dewar HA. An epidemic of mussel poisoning in North-East England. *Lancet* 1968; **2**(7571): 767-70.
8. Meinke III AH, Quinn EL. Paralytic shellfish poisoning. *Mich Med* 1973; **72**(1): 37-8.
9. Ayres PA. Mussel poisoning in Britain with special reference to paralytic shellfish poisoning. A review of cases reported 1814-1968. *Envir Hlth* 1975; **83**(7): 261-5.
10. Fortuine R. Paralytic shellfish poisoning in the North Pacific: two historical accounts and implications for today. *Alaska Med* 1975; **17**(5): 71-5.
11. Rhodes FA, Mills CG, Popei K. Paralytic shellfish poisoning in Papua New Guinea. *P N G Med J* 1975; **18**(4): 197-202.
12. Blanc MH, Zwahlen A, Robert M. Symptoms of shellfish poisoning. *N Engl J Med* 1977; **296**(5): 287-8.
13. Cladouhos JW. Paralytic shellfish poisonings reported in Alaska. *J Environ Health* 1977; **39**(4): 256-7.
14. Craun GF. Waterborne outbreaks. *J Water Pollut Control Fed* 1977; **49**(6): 1268-79.
15. Hughes JM, Horwitz MA, Merson MH, Barker WH, Jr., Gangarosa EJ. Foodborne disease outbreaks of chemical etiology in the United States, 1970-1974. *Am J Epidemiol* 1977; **105**(3): 233-44.
16. Morse EV. Paralytic shellfish poisoning: a review. *J Am Vet Med Assoc* 1977; **171**(11): 1178-80.
17. Roy RN. Red tide and outbreak of paralytic shellfish poisoning in Sabah. *Med J Malaysia* 1977; **31**(3): 247-51.
18. Todd ECD. Foodborne Disease in Canada - 1974 Annual Summary. *J Food Prot* 1977; **40**(7): 493-8.
19. Zwahlen A, Blanc MH, Robert M. ["Paralytic shellfish poisoning" (author's transl)]. *Schweiz Med Wochenschr* 1977; **107**(7): 226-30.
20. Acres J, Gray J. Paralytic shellfish poisoning. *CMAJ* 1978; **119**(10): 1195-7.
21. Blanc MH, Zwahlen A, Robert M. [Epidemic of mussel poisoning]. *Rev Clin Esp* 1978; **150**(5): 259-63.
22. Caroli G, Malfatti S, Armani G. An epidemic of neurotoxic shellfish poisoning due to imported mussels. *Riv Ital Ig* 1978; **38**(1-2): 3-11.
23. Imbert JC, Essaïd el Feydi A, Kadiri A. [Paralytic shellfish poisoning (author's transl)]. *Sem Hop* 1979; **55**(21-22): 1139-42.

24. Popkiss ME, Horstman DA, Harpur D. Paralytic shellfish poisoning. A report of 17 cases in Cape Town. *S Afr Med J* 1979; **55**(25): 1017-23.
25. Bryan FL. Epidemiology of Foodborne Diseases Transmitted by Fish, Shellfish and Marine Crustaceans in the United States, 1970-1978. *J Food Prot* 1980; **43**(11): 859-76.
26. Grimard D, Lalonde R. [Paralysing shell fish poisoning]. *Union Med Can* 1981; **110**(2): 144-50.
27. Gulbrandsen RK, Aalvik B. [Shellfish poisoning. A short review of shellfish poisoning and a description of an outbreak]. *Tidsskr Nor Laegeforen* 1981; **101**(7): 452-4.
28. Todd ECD. Foodborne and Waterborne Disease in Canada - 1977 Annual Summary. *J Food Prot* 1982; **45**(9): 865-73.
29. de la Garza Aguilar J. [Food poisoning caused by ingestion of contaminated shellfish]. *Salud Publica Mex* 1983; **25**(2): 145-50.
30. None Listed. Leads from the MMWR. Annual mussel quarantine--California, 1983. *JAMA* 1983; **249**(24): 3292.
31. Tangen K. Shellfish poisoning and the occurrence of potentially toxic dinoflagellates in norwegian waters. *Sarsia* 1983; **68**(1): 1-7.
32. Conte FS. Economic impact of paralytic shellfish poison on the oyster industry in the Pacific United States. *Aquaculture* 1984; **39**(1-4): 331-43.
33. Hasselgård T, Hjelle A. [Shellfish poisoning. An epidemic in the Nesset district]. *Tidsskr Nor Laegeforen* 1984; **104**(5): 292-4.
34. Langeland G, Hasselgård T, Tangen K, Skulberg OM, Hjelle A. An outbreak of paralytic shellfish poisoning in Western Norway. *Sarsia* 1984; **69**(3-4): 185-93.
35. Gacutan RQ, Tabbu MY, Aujero EJ, Icatlo Jr F. Paralytic shellfish poisoning due to Pyrodinium bahamense var. compressa in Mati, Davao Oriental, Philippines. *Mar Biol* 1985; **87**(3): 223-7.
36. Todd ECD. Foodborne and Waterborne Disease in Canada - 1978 Annual Summary. *J Food Prot* 1985; **48**(11): 990-6.
37. Todd ECD. Foodborne and Waterborne Disease in Canada - 1979 Annual Summary. *J Food Prot* 1985; **48**(12): 1071-8.
38. Kan SK, Singh N, Chan MK. Oliva vidua fulminans, a marine mollusc, responsible for five fatal cases of neurotoxic food poisoning in Sabah, Malaysia. *Trans R Soc Trop Med Hyg* 1986; **80**(1): 64-5.
39. Kim JY, Lee CU, Jun JH. An epidemiologic study of paralytic shellfish poisoning. *Taehan Uihak Hyophoe Chi* 1986; **29**(8): 896-905.
40. Mee LD, Espinosa M, Diaz G. Paralytic shellfish poisoning with a Gymnodinium catenatum red tide on the Pacific Coast of Mexico. *Mar Environ Res* 1986; **19**(1): 77-92.
41. Vecchio JH, Gómez O, Orosco JA, Tartaglione JC, Gricman G. Poisoning by paralysing mollusk venom (red tide). *Medicina* 1986; **46**(6): 705-8.
42. Eason RJ, Harding E. Neurotoxic fish poisoning in the Solomon Islands. *P N G Med J* 1987; **30**(1): 49-52.
43. Sanders WE, Jr. Intoxications from the seas: ciguatera, scombroid, and paralytic shellfish poisoning. *Infect Dis Clin North Am* 1987; **1**(3): 665-76.
44. Todd ECD. Foodborne and Waterborne Disease in Canada - 1980 Annual Summary. *J Food Prot* 1987; **50**(5): 420-8.
45. Mills AR, Passmore R. Pelagic paralysis. *Lancet* 1988; **1**(8578): 161-4.

46. None Listed. Illness due to molluscan shellfish--England and Wales. *Can Dis Wkly Rep* 1988; **14**(13): 55-6.
47. Todd ECD. Foodborne and Waterborne Disease in Canada - 1982 Annual Summary. *J Food Prot* 1988; **51**(1): 56-65.
48. Hashimoto K, Noguchi T. Recent studies on paralytic shellfish poison in Japan. *Pure and Applied Chemistry (PAC)* 1989; **61**(1): 7-18.
49. Maclean JL. Indo-Pacific red tides, 1985-1988. *Mar Pollut Bull* 1989; **20**(7): 304-10.
50. Swaddiwudhipong W, Kunasol P, Sangwanloy O, Srisomporn D. Foodborne disease outbreaks of chemical etiology in Thailand, 1981-1987. *Southeast Asian J Trop Med Public Health* 1989; **20**(1): 125-32.
51. Todd ECD. Foodborne and Waterborne Disease in Canada - 1983 Annual Summary. *J Food Prot* 1989; **52**(6): 436-42.
52. Long RR, Sargent JC, Hammer K. Paralytic shellfish poisoning: A case report and serial electrophysiologic observations. *Neurology* 1990; **40**(8): 1310-2.
53. Mata L, Abarca G, Marranghello L, Viquez R. Paralytic Shellfish Poisoning (Pdsp) Due to Spondylus-Calcifer Contaminated by Pyrodinium-Bahamense, Costa-Rica, 1989-1990. *Rev Biol Trop* 1990; **38**(1): 129-36.
54. None Listed. Paralytic shellfish poisoning (red tide). *Epidemiol Bull* 1990; **11**(2): 9.
55. Rodrigue DC, Etzel RA, Hall S, et al. Lethal paralytic shellfish poisoning in Guatemala. *Am J Trop Med Hyg* 1990; **42**(3): 267-71.
56. The PHLS Communicable Disease Surveillance Centre. Communicable disease report January to June 1990. *J Public Health* 1990; **12**(3-4): 209-12.
57. Ahmed FE. Naturally Occurring Seafood Toxins. *Journal of Toxicology: Toxin Reviews* 1991; **10**(3): 263-87.
58. Cheng HS, Chua SO, Hung JS, Yip KK. Creatine kinase MB elevation in paralytic shellfish poisoning. *Chest* 1991; **99**(4): 1032-3.
59. None Listed. Food safety. Paralytic shellfish poisoning. *Wkly Epidemiol Rec* 1991; **66**(25): 185-7.
60. Saldate Castañeda O, Vázquez Castellanos JL, Galván J, Sánchez Anguiano A, Nazar A. [Poisoning by paralytic molluscan toxins in Oaxaca]. *Salud Publica Mex* 1991; **33**(3): 240-7.
61. Scoging AC. Illness associated with seafood. *CDR (Lond Engl Rev)* 1991; **1**(11): R117-22.
62. United States Centers for Disease Control and Prevention. Paralytic shellfish poisoning--Massachusetts and Alaska, 1990. *MMWR Morb Mortal Wkly Rep* 1991; **40**(10): 157-61.
63. Ahmed FE. Review: Assessing and managing risk due to consumption of seafood contaminated with micro-organisms, parasites, and natural toxins in the US. *Int J Food Sci Technol* 1992; **27**(3): 243-60.
64. Viviani R. Eutrophication, marine biotoxins, human health. *Sci Total Environ* 1992; **Suppl**: 631-62.
65. Montebruno D. [Poisoning by the consumption of shellfish contaminated with paralytic venom in the XII Region, Chile. Anatomopathological study]. *Rev Med Chil* 1993; **121**(1): 94-7.
66. Montebruno D. Paralytic shellfish poisoning in Chile. *Med Sci Law* 1993; **33**(3): 243-6.
67. Moss ML. Shellfish, Gender, and Status on the Northwest Coast - Reconciling Archaeological, Ethnographic, and Ethnohistorical Records of the Tlingit. *Am Anthropol* 1993; **95**(3): 631-52.

68. Negoro K, Morimatsu M. [Clinical analysis of paralytic shellfish poisoning following ingestion of oysters]. *Rinsho Shinkeigaku* 1993; **33**(2): 207-9.
69. Saavedra-Deigado AM, Metcalfe DD. Seafood toxins. *Clin Rev Allergy* 1993; **11**(2): 241-60.
70. Todd E, Avery G, Grant GA, Fenwick JC, Chiang R, Babiuk T. An outbreak of severe paralytic shellfish poisoning in British Columbia. *Can Commun Dis Rep* 1993; **19**(13): 99-102.
71. Hartigan-Go K, Bateman DN. Redtide in the Philippines. *Hum Exp Toxicol* 1994; **13**(12): 824-30.
72. Ledoux MCNdEVeA, Paris (France). Laboratoire Central d'Hygiene Alimantaire), Fremy JM. Phytoplankton, phycotoxins and seafood poisoning. *Rec Med Vet Ec Alfort* 1994; **v. 170**.
73. Todd EC. Emerging diseases associated with seafood toxins and other water-borne agents. *Ann N Y Acad Sci* 1994; **740**: 77-94.
74. Corrales RA, Maclean JL. Impacts of harmful algae on seafarming in the Asia-Pacific areas. *J Appl Phycol* 1995; **7**(2): 151-62.
75. Cortés-Altamirano R, Hernandez-Becerril DU, Luna-Soria R. [Red tides in Mexico: a review]. *Rev Latinoam Microbiol* 1995; **37**(4): 343-52.
76. Gessner BD, Middaugh JP. Paralytic shellfish poisoning in Alaska: a 20-year retrospective analysis. *Am J Epidemiol* 1995; **141**(8): 766-70.
77. Hwang DF, Tsai YH, Cheng CA, et al. 2 Food Poisoning Incidents Due to Ingesting the Purple Clam Occurred in Taiwan. *J Nat Toxins* 1995; **4**(2): 173-9.
78. Bean NH, Goulding JS, Lao C, Angulo FJ. Surveillance for foodborne-disease outbreaks--United States, 1988-1992. *MMWR CDC Surveill Summ* 1996; **45**(5): 1-66.
79. Gessner BD, Schloss M. A population-based study of paralytic shell fish poisoning in Alaska. *Alaska Med* 1996; **38**(2): 54-8, 68.
80. Martin R, Garcia T, Sanz B, Hernandez PE. Seafood toxins: Poisoning by bivalve consumption. *Food Sci Technol Int* 1996; **2**(1): 13-22.
81. Bean NH, Goulding JS, Daniels MT, Angulo FJ. Surveillance for Foodborne Disease Outbreaks-United States, 1988-1992. *J Food Prot* 1997; **60**(10): 1265-86.
82. Gessner BD, Bell P, Doucette GJ, et al. Hypertension and identification of toxin in human urine and serum following a cluster of mussel-associated paralytic shellfish poisoning outbreaks. *Toxicon* 1997; **35**(5): 711-22.
83. Gessner BD, Middaugh JP, Doucette GJ. Paralytic shellfish poisoning in Kodiak, Alaska. *West J Med* 1997; **167**(5): 351-3.
84. Todd EC. Seafood-associated diseases and control in Canada. *Rev Sci Tech* 1997; **16**(2): 661-72.
85. Akaeda H, Takatani T, Anami A, Noguchi T. Mass outbreak of paralytic shellfish poisoning due to ingestion of oysters at Tamano-ura, Goto Islands, Nagasaki, Japan. *Shokuhin Eiseigaku Zasshi* 1998; **39**(4): 272-4.
86. de Carvalho M, Jacinto J, Ramos N, de Oliveira V, Pinho e Melo T, de Sá J. Paralytic shellfish poisoning: clinical and electrophysiological observations. *J Neurol* 1998; **245**(8): 551-4.
87. Scoging AC. Marine biotoxins. *Symp Ser Soc Appl Microbiol* 1998; **27**: 41s-50s.
88. Sierra-Beltrán AP, Cruz A, Núñez E, Del Villar LM, Cerecero J, Ochoa JL. An overview of the marine food poisoning in Mexico. *Toxicon* 1998; **36**(11): 1493-502.

89. Trevino S. Fish and shellfish poisoning. *Clin Lab Sci* 1998; **11**(5): 309-14.
90. Bankoff G. Societies in conflict: algae and humanity in the Philippines. *Env Hist Camb* 1999; **5**(1): 97-123.
91. Morris Jr JG. Harmful algal blooms: An emerging public health problem with possible links to human stress on the environment. *Annual Review of Energy and the Environment* 1999; **24**: 367-90.
92. Murakami R, Noguchi T. Paralytic shellfish poison. *Shokuhin Eiseigaku Zasshi* 2000; **41**(1): 1-10.
93. Azanza RV, Taylor FJR. Are Pyrodinium blooms in the Southeast Asian region recurring and spreading? A view at the end of the millennium. *Ambio* 2001; **30**(6): 356-64.
94. Lehane L. Paralytic shellfish poisoning: a potential public health problem. *Med J Aust* 2001; **175**(1): 29-31.
95. Bajarias FFA, Furio EF, Gonzales CL, Sakamoto S, Fukuyo Y, Kodama M. Localizing PSP Monitoring in the Philippines: A management option. *Fish Sci* 2002; **68**: 519-22.
96. Holmes MJ, Teo SLM. Toxic marine dinoflagellates in Singapore waters that cause seafood poisonings. *Clin Exp Pharmacol Physiol* 2002; **29**(9): 829-36.
97. Krys S, Frémy JM. Phycotoxins and seafoods: Associated medical risks and preventive measures. *Revue Francaise des Laboratoires* 2002; **2002**(348): 29-38.
98. Marcus. Neurologic illness associated with eating Florida pufferfish, 2002. *MMWR Morb Mortal Wkly Rep* 2002; **51**(15): 321-3.
99. United States Centers for Disease Control and Prevention. Update: Neurologic illness associated with eating Florida pufferfish, 2002. *MMWR Morb Mortal Wkly Rep* 2002; **51**(19): 414-6.
100. Balmer-Hanchey E, Jaykus L-A, McClellan-Green P. Marine Biotoxins of Algal Origin and Seafood Safety. *J Aquat Food Prod* 2003; **12**: 29-53.
101. Barbier HM, Diaz JH. Prevention and treatment of toxic seafoodborne diseases in travelers. *J Travel Med* 2003; **10**(1): 29-37.
102. García C, del Carmen Bravo M, Lagos M, Lagos N. Paralytic shellfish poisoning: post-mortem analysis of tissue and body fluid samples from human victims in the Patagonia fjords. *Toxicon* 2004; **43**(2): 149-58.
103. La Barbera-Sánchez A, Franco Soler J, Rojas de Astudillo L, Chang-Yen I. Paralytic shellfish poisoning (PSP) in Margarita Island, Venezuela. *Rev Biol Trop* 2004; **52 Suppl 1**: 89-98.
104. Batoréu MC, Dias E, Pereira P, Franca S. Risk of human exposure to paralytic toxins of algal origin. *Environ Toxicol Pharmacol* 2005; **19**(3): 401-6.
105. García C, Lagos M, Truan D, et al. Human intoxication with paralytic shellfish toxins: clinical parameters and toxin analysis in plasma and urine. *Biol Res* 2005; **38**(2-3): 197-205.
106. Hernández C, Ulloa J, Vergara JA, Espejo R, Cabello F. [Vibrio parahaemolyticus infections and algal intoxications as emergent public health problems in Chile]. *Rev Med Chil* 2005; **133**(9): 1081-8.
107. Mira Gutiérrez J. [The man and the sea: marine phytoplancton and public health]. *An R Acad Nac Med (Madr)* 2005; **122**(4): 661-79; discussion 79.
108. Rapala J, Robertson A, Negri AP, et al. First report of saxitoxin in Finnish lakes and possible associated effects on human health. *Environ Toxicol* 2005; **20**(3): 331-40.
109. Sobel J, Painter J. Illnesses caused by marine toxins. *Clin Infect Dis* 2005; **41**(9): 1290-6.

110. Azanza MPV. Philippine foodborne-disease outbreaks (1995-2004). *J Food Saf* 2006; **26**(1): 92-102.
111. Chung PH, Chuang SK, Tsang T. Consumption of viscera as the most important risk factor in the largest outbreak of shellfish poisoning in Hong Kong, 2005. *Southeast Asian J Trop Med Public Health* 2006; **37**(1): 120-5.
112. Fortuine R. Paralytic shellfish poisoning in the north Pacific: two historical accounts and implications for today. 1975. *Alaska Med* 2007; **49**(2): 65-9.
113. Jen HC, Yen JY, Liao IC, Hwang DF. Identification of Species and Paralytic Shellfish Poisons in an Unknown Scallop Meat Implicated in Food Poisoning in Taiwan. *Raffles Bulletin of Zoology (RBZ)* 2008: 115-22.
114. Wang DZ. Neurotoxins from marine dinoflagellates: a brief review. *Mar Drugs* 2008; **6**(2): 349-71.
115. Barraza JE. Food poisoning due to consumption of the marine gastropod Plicopurpura columellaris in El Salvador. *Toxicon* 2009; **54**(6): 895-6.
116. James KJ, Carey B, O'Halloran J, van Pelt FN, Skrabáková Z. Shellfish toxicity: human health implications of marine algal toxins. *Epidemiol Infect* 2010; **138**(7): 927-40.
117. Bienfang PK, Defelice SV, Laws EA, et al. Prominent human health impacts from several marine microbes: history, ecology, and public health implications. *Int J Microbiol* 2011; **2011**: 152815.
118. Hinder SL, Hays GC, Brooks CJ, et al. Toxic marine microalgae and shellfish poisoning in the British isles: history, review of epidemiology, and future implications. *Environ Health* 2011; **10**: 54.
119. McLaughlin JB, Fearey DA, Esposito TA, Porter KA. Paralytic shellfish poisoning - southeast Alaska, may-june 2011. *MMWR Morb Mortal Wkly Rep* 2011; **60**(45): 1554-6.
120. Toda M, Uneyama C, Toyofuku H, Morikawa K. [Trends of food poisonings caused by natural toxins in Japan, 1989-2011]. *Shokuhin Eiseigaku Zasshi* 2012; **53**(2): 105-20.
121. Gould LH, Walsh KA, Vieira AR, et al. Surveillance for foodborne disease outbreaks - United States, 1998-2008. *MMWR Surveill Summ* 2013; **62**(1).
122. Turnbull A, Harrison R, McKeown S. Paralytic shellfish poisoning in south eastern Tasmania. *Commun Dis Intell Q Rep* 2013; **37**(1): E52-4.
123. DeGrasse S, Rivera V, Roach J, et al. Paralytic shellfish toxins in clinical matrices: Extension of AOAC official method 2005.06 to human urine and serum and application to a 2007 case study in Maine. *Deep Sea Res 2 Top Stud Oceanogr* 2014; **103**: 368-75.
124. Hurley W, Wolterstorff C, MacDonald R, Schultz D. Paralytic shellfish poisoning: a case series. *West J Emerg Med* 2014; **15**(4): 378-81.
125. Trainer VL, Sullivan K, Eberhart B-TL, et al. Enhancing Shellfish Safety in Alaska through Monitoring of Harmful Algae and Their Toxins. *J Shellfish Res* 2014; **33**(2): 531-9.
126. Callejas L, Darce AC, Amador JJ, et al. Paralytic shellfish poisonings resulting from an algal bloom in Nicaragua. *BMC Res Notes* 2015; **8**: 74.
127. Ching PK, Ramos RA, de los Reyes VC, Sucaldito MN, Tayag E. Lethal paralytic shellfish poisoning from consumption of green mussel broth, Western Samar, Philippines, August 2013. *Western Pac Surveill Response J* 2015; **6**(2): 22-6.
128. Clemence MA, Guerrant RL. Infections and intoxications from the ocean: Risks of the shore. *Microbiol Spectr* 2015; **3**(6).
129. Knaack JS, Porter KA, Jacob JT, et al. Case diagnosis and characterization of suspected paralytic shellfish poisoning in Alaska. *Harmful Algae* 2016; **57**(Pt B): 45-50.

130. Suleiman M, Jelip J, Rundi C, Chua TH. Case Report: Paralytic Shellfish Poisoning in Sabah, Malaysia. *Am J Trop Med Hyg* 2017; **97**(6): 1731-6.
131. Arnich N, Thebault A. Dose-Response Modelling of Paralytic Shellfish Poisoning (PSP) in Humans. *Toxins (Basel)* 2018; **10**(4).
132. Coleman RM, Ojeda-Torres G, Bragg W, et al. Saxitoxin Exposure Confirmed by Human Urine and Food Analysis. *J Anal Toxicol* 2018; **42**(7): e61-e4.
133. Edwards LJ, Wilson K, Veitch MG. An Outbreak of Paralytic Shellfish Poisoning in Tasmania. *Commun Dis Intell (2018)* 2018; **42**.
134. Carvalho ILD, Pelerito A, Ribeiro I, Cordeiro R, Núncio MS, Vale P. Paralytic shellfish poisoning due to ingestion of contaminated mussels: A 2018 case report in Caparica (Portugal). *Toxicon: X* 2019; **4**.
135. Marks CJ, Van Hoving DJ, Wium CA, et al. South African Marine Envenomations and Poisonings as Managed Telephonically by the Tygerberg Poisons Information Centre: A 20-Year Retrospective Review. *Wilderness Environ Med* 2019; **30**(2): 134-40.
136. Azzeri A, Ching GH, Jaafar H, et al. A Review of Published Literature Regarding Health Issues of Coastal Communities in Sabah, Malaysia. *Int J Environ Res Public Health* 2020; **17**(5).
137. Vale P. Shellfish contamination with marine biotoxins in Portugal and spring tides: a dangerous health coincidence. *Environ Sci Pollut Res Int* 2020; **27**(33): 41143-56.
138. Anderson DM, Fensin E, Gobler CJ, et al. Marine harmful algal blooms (HABs) in the United States: History, current status and future trends. *Harmful Algae* 2021; **102**.
139. Karlson B, Andersen P, Arneborg L, et al. Harmful algal blooms and their effects in coastal seas of Northern Europe. *Harmful Algae* 2021; **102**.
140. McIntyre L, Miller A, Kosatsky T. Changing Trends in Paralytic Shellfish Poisonings Reflect Increasing Sea Surface Temperatures and Practices of Indigenous and Recreational Harvesters in British Columbia, Canada. *Mar Drugs* 2021; **19**(10).
141. Sunesen I, Mendez SM, Mancera-Pineda JE, Bottein MYD, Enevoldsen H. The Latin America and Caribbean HAB status report based on OBIS and HAEDAT maps and databases. *Harmful Algae* 2021; **102**.
142. Velayudhan A, Nayak J, Murhekar MV, Dikid T, Sodha SV. Shellfish poisoning outbreaks in Cuddalore District, Tamil Nadu, India. *Indian J Public Health* 2021; **65**(Supplement): S29-s33.
143. Barría C, Vásquez-Calderón P, Lizama C, et al. Spatial Temporal Expansion of Harmful Algal Blooms in Chile: A Review of 65 Years Records. *J Mar Sci Eng* 2022; **10**(12): 1868.
144. Sinno-Tellier S, Abadie E, de Haro L, et al. Human poisonings by neurotoxic phycotoxins related to the consumption of shellfish: study of cases registered by the French Poison Control Centres from 2012 to 2019. *Clin Toxicol (Phila)* 2022; **60**(6): 759-67.
145. Temple C, Hughes A. A case of fatal paralytic shellfish poisoning in Alaska. *Clin Toxicol (Phila)* 2022; **60**(3): 414-5.
146. Chen J, Hong S, Zhang J, Cai. M, Lin Y. Investigation into an outbreak of suspected shellfish poisoning caused by consuming Bullacta exarata. *Chinese Journal of Food Hygiene* 2023; **35**(8): 1231-4.
147. Mafra Jr. LL, Sunesen I, Pires E, et al. Benthic harmful microalgae and their impacts in South America. *Harmful Algae* 2023; **127**: 102478.
148. Reséndiz-Colorado G, García-Mendoza E, Almazán-Becerril A, Medina-Elizalde J, Cepeda-Morales JA, Rivera-Caicedo JP. Towards the Early Detection of Gymnodinium

catenatum Algal Blooms in the Northern Gulf of California. *Journal of Marine Science and Engineering,* 2023. (accessed.
149. Sinno-Tellier S, Abadie E, Guillotin S, Bossée A, Nicolas M, Delcourt N. Human shellfish poisoning: Implementation of a national surveillance program in France. *Front Mar Sci* 2023; **9**(2022): 1089585.
150. Yu Z, Tang Y, Gobler CJ. Harmful algal blooms in China: History, recent expansion, current status, and future prospects. *Harmful Algae* 2023; **129**: 102499.
151. Zheng R, Huang L, Wu Y, Lin S, Huang L. Simultaneous analysis of paralytic shellfish toxins and tetrodotoxins in human serum by liquid chromatography coupled to Q-Exactive high-resolution mass spectrometry. *J Chromatogr B* 2023; **1215**: 123565.
152. Rodríguez F, Escalera L, Reguera B, et al. Red tides in the Galician rías: historical overview, ecological impact, and future monitoring strategies. *Environmental Science: Processes & Impacts* 2024; **26**(1): 16-34.

Table S2. Outbreak Report Details. Articles are grouped by continent and in alphabetical order. Each article's language, number of outbreaks reported, and exposure location is provided.

Continent	First Author, Year	Language	Outbreak Count	Exposure Location		
				Home	Vacation or Tourists	Not Reported
Africa	McFarren, 1960	English	≥1 (>44)*			X
	Popkiss, 1979	English	1	X		X
	Batoréu, 2005	English	1 (8)*			X
	Arnich, 2018	English	1 (14)*			X
	Marks, 2019	English	2			X
	Vale, 2020	English	1 (4)*	X		X
Asia	Kawabata, 1962	English	1			X
	Roy, 1977	English	3	X		
	Imbert, 1979	French	44*			X
	Gacutan, 1985	English	1			X
	Kan, 1986	English	1	X		
	Kim, 1986	Korean[†]	1			X
	Hashimoto, 1989	English	7			X
	MacLean, 1989	English	≥9 (≥11)*			X
	Swaddiwudhipong, 1989	English	1			X
	Cheng, 1991	English	1			X

Continent	First Author, Year	Language	Outbreak Count	Exposure Location		
				Home	Vacation or Tourists	Not Reported
Asia (cont.)	Viviani, 1992	English	≥1 (>4)*			X
	Negoro, 1993	Japanese	1	X		
	Hartigan-Go, 1994	English	4	X		X
	Todd, 1994	English	1			X
	Corrales, 1995	English	1			X
	Hwang, 1995	English	2			X
	Akaeda, 1998	English	1	X		
	Trevino, 1998	English	14*			X
	Bankoff, 1999	English	7 (9)*			X
	Morris Jr., 1999	English	52*			X
	Murakami, 2000	Japanese	12			X
	Azanza, 2001	English	14			X
	Bajarias, 2002	English	1			X
	Holmes, 2002	English	1			X
	Batoréu, 2005	English	1 (8)*			X
	Azanza, 2006	English	1			X
	Chung, 2006	English	1			X

Continent	First Author, Year	Language	Outbreak Count	Exposure Location		
				Home	Vacation or Tourists	Not Reported
Asia (cont.)	Jen, 2008	English	1			X
	James, 2010	English	1 (10)*			X
	Toda, 2012	Japanese	8			X
	Ching, 2015	English	1	X		
	Suleiman, 2017	English	4	X	X	
	Arnich, 2018	English	1 (14)*			X
	Azzeri, 2020	English	1			X
	Velayudhan, 2021	English	2	X		
	Chen, 2023	Mandarin (Chinese)	1	X		
	Yu, 2023	English	1			X
	Zheng, 2023	English	1			X
Europe	McFarren, 1960	English	≥13 (>44)*			X
	McCollum, 1968	English	1	X		
	Ayres, 1975	English	10			X
	Blanc, 1977	English	1			X
	Zwahlen, 1977	French	1	X		
	Blanc, 1978	Spanish	1	X		
	Caroli, 1978	Italian	1	X		
	Imbert, 1979	French	44*			X

Continent	First Author, Year	Language	Outbreak Count	Exposure Location		
				Home	Vacation or Tourists	Not Reported
Europe (cont.)	Gulbrandsen, 1981	Norwegian	1	X		
	Tangen, 1983	English	1		X	
	Hasselgård, 1984	Norwegian	1	X		
	Langeland, 1984	English	1			X
	Sanders, 1987	English	Not Listed*			X
	Mills, 1988	English	≥2 (>5)*			X
	None Listed, 1988	English; French	4 (5)*	X		
	The PHLS Communicable Disease Surveillance Centre, 1990	English	1			X
	Scoging, 1991	English	10			X
	Viviani, 1992	English	>2 (>4)*			X
	Ledoux, 1994	French	2			X
	Martin, 1996	Spanish	1			X
	de Carvalho, 1998	English	1			X
	Scoging, 1998	English	10			X
	Krys, 2002	French	>5			X
	Batoréu, 2005	English	2 (8)*			X
	Mira Gutiérrez, 2005	Spanish	7			X

Continent	First Author, Year	Language	Outbreak Count	Exposure Location		
				Home	Vacation or Tourists	Not Reported
Europe (cont.)	Rapala, 2005	English	1	X		
	James, 2010	English	4 (10)*			X
	Hinder, 2011	English	1			X
	Arnich, 2018	English	2 (14)*			X
	Carvalho, 2019	English	1			X
	Vale, 2020	English	3 (4)*	X		X
	Karlson, 2021	English	1			X
	Sinno-Tellier, 2022	English	15			X
	Rodríguez, 2024	English	1			X
North America	Scobey, 1947	English	1			X
	Meyers, 1955	English	1	X		
	Tennant, 1955	English	1		X	
	Bond, 1958	English	2	X	X	
	McFarren, 1960	English	≥29 (>44)*			X
	Meinke 3rd, 1973	English	1			X
	Fortuine, 1975	English	2		X	
	Cladouhos, 1977	English	1	X	X	
	Craun, 1977	English	2			X

Continent	First Author, Year	Language	Outbreak Count	Exposure Location		
				Home	Vacation or Tourists	Not Reported
North America (cont.)	Hughes, 1977	English	6			X
	Morse, 1977	English	8			X
	Todd, 1977	English	1			X
	Acres, 1978	English	1		X	
	Imbert, 1979	French	44*			X
	Bryan, 1980	English	14			X
	Grimard, 1981	French	2	X		
	Todd, 1982	English	2			X
	de la Garza Aguilar, 1983	Spanish	1			X
	None Listed, 1983	English	1			X
	Conte, 1984	English	13			X
	Todd, 1985a	English	2			X
	Todd, 1985b	English	2			X
	Mee, 1986	English	1	X		
	Sanders, 1987	English	20*			X
	Todd, 1987	English	2			X
	Mills, 1988	English	≥3 (>5)*			X

Continent	First Author, Year	Language	Outbreak Count	Exposure Location		
				**Home**	**Vacation or Tourists**	**Not Reported**
**North America (cont.)**	None Listed, 1988	English; French	1 (5)*	**X**		
	Todd, 1988	English	2			**X**
	Todd, 1989	English	1			**X**
	Long, 1990	English	1			**X**
	Mata, 1990	Spanish	1	**X**		
	None Listed, 1990	English	3			**X**
	Rodrigue, 1990	English	1			**X**
	Ahmed, 1991	English	13			**X**
	None Listed, 1991	English; French	1			**X**
	Saldate Castañeda, 1991	Spanish	1			**X**
	United States Centers for Disease Control and Prevention, 1991	English	2	**X**		
	Ahmed, 1992	English	1			**X**
	Viviani, 1992	English	≥1 (>4)*			**X**
	Moss, 1993	English	2		**X**	
	Saaverda-Deigado, 1993	English	14			**X**
	Todd, 1993	English; French	1	**X**		
	Cortés-Altamirano, 1995	Spanish	6			**X**
	Gessner, 1995	English	1			**X**

Continent	First Author, Year	Language	Outbreak Count	Exposure Location		
				Home	Vacation or Tourists	Not Reported
North America (cont.)	Bean, 1996	English	3			X
	Gessner, 1996	English	1			X
	Bean, 1997	English	5	X		X
	Gessner, 1997a	English	4			X
	Gessner, 1997b	English	1	X		
	Todd, 1997	English	11			X
	Sierra-Beltrán, 1998	English	11			X
	Trevino, 1998	English	14*			X
	Bankoff, 1999	English	1 (9)*			X
	Morris Jr., 1999	English	52*			X
	United States Centers for Disease Control and Prevention, 2002a	English	1			X
	United States Centers for Disease Control and Prevention, 2002b	English	3			X
	Balmer-Hanchey, 2003	English	9			X
	Barbier, 2003	English	13			X
	Batoréu, 2005	English	2 (8)*			X
	Sobel, 2005	English	8			X
	Fortuine, 2007	English	6	X		

Continent	First Author, Year	Language	Outbreak Count	Exposure Location		
				Home	Vacation or Tourists	Not Reported
North America (cont.)	Wang, 2008	English	1			X
	Barraza, 2009	English	1			X
	James, 2010	English	4 (10)*			X
	Bienfang, 2011	English	1			X
	McLaughlin, 2011	English	1			X
	Gould, 2013	English	1	X		
	DeGrasse, 2014	English	1	X		
	Hurley, 2014	English	1		X	
	Trainer, 2014	English	1			X
	Callejas, 2015	English	1	X		
	Clemence, 2015	English	65			X
	Knaack, 2016	English	1	X		
	Arnich, 2018	English	8 (14)*			X
	Coleman, 2018	English	1	X		
	Anderson, 2021	English	3			X
	McIntyre, 2021	English	62	X	X	
	Sunesen, 2021	English	6 (14)*			X
	Temple, 2022	English	1	X		
	Reséndiz-Colorado, 2023	English	1			X
Oceania	McFarren, 1960	English	≥1 (>44)*			X
	Rhodes, 1975	English	1	X	X	
	Eason, 1987	English	2	X		

Continent	First Author, Year	Language	Outbreak Count	Exposure Location		
				Home	Vacation or Tourists	Not Reported
Oceania (cont.)	MacLean, 1989	English	≥2 (>11)*			X
	Bankoff, 1999	English	1 (9)*			X
	Lehane, 2001	English	Unspecified			X
	Turnbull, 2013	English	1			X
	Arnich, 2018	English	1 (14)*			X
	Edwards, 2018	English	1	X		
South America	Vecchio, 1986	Spanish	1			X
	Montebruno, 1993a	English	1			X
	Montebruno, 1993b	Spanish	1			X
	García, 2004	English	1	X		
	La Barbera-Sánchez, 2004	English	1	X		
	Batoréu, 2005	English	1 (8)*			X
	García, 2005	English	1			X
	Hernández, 2005	Spanish	1			X
	James, 2010	English	1 (10)*			X
	Arnich, 2018	English	1 (14)*			X
	Sunesen, 2021	English	8 (14)*			X
	Barría, 2022	English	25			X
	Mafra Jr., 2023	English	3			X
Global	Lehane, 2001	English	Unspecified			X
	Sinno-Teller, 2023	English	Unspecified			X

[†]*This article was translated into English.*
[*]*Outbreak count was not stratified by continent.*

Table S3. Quality of the Evidence and Risk of Bias. "Other" under study design include case-control studies and chemical analyses with outbreak data. Case definition was defined as "Definite" (symptoms attributed to PSP after seafood eaten AND saxitoxin biomarker confirmed - human urine or in seafood eaten), "Probable" (symptoms attributed to PSP after seafood eaten but NO saxitoxin biomarker confirmation), "Possible" (symptoms noted as possibly consistent with PSP after seafood eaten and NO saxitoxin biomarker confirmation), or "Not Specified" (e.g., surveillance reports with total PSP numbers in a decade). Author institutional affiliations were defined for all coauthors, not just the first author.

Continent	First Author, Year	Study Design					Case Definition				Author Institutional Affiliation			Author Departmental Affiliation			Funding/ Sponsorship Source				Generalizability
		Ecologic or Time-series or Surveillance	Case series	Case Report	Literature Review	Other	Definite	Probable	Possible	Not Specified	University or Academic Institution	Government	Other	Medicine or Public Health	Ecology or Environmental Sciences	Other	Government	Non-Government	Unsponsored	Not Reported	
Africa	McFarren, 1960				X					X		X		X		X				X	
	Popkiss, 1979	X						X			X			X	X					X	
	Batoréu, 2005		X					X			X			X						X	
	Arnich, 2018				X					X		X		X			X				
	Marks, 2019	X								X	X			X						X	
	Vale, 2020		X				X					X			X		X				
Asia	Kawabata, 1962		X							X	X	X		X						X	
	Roy, 1977		X				X						X	X						X	
	Imbert, 1979	X								X	X	X				X				X	
	Gacutan, 1985			X				X				X	X	X	X		X				

Continent	First Author, Year	Study Design					Case Definition				Author Institutional Affiliation			Author Departmental Affiliation			Funding/ Sponsorship Source				Generalizability
		Ecologic or Time-series or Surveillance	Case series	Case Report	Literature Review	Other	Definite	Probable	Possible	Not Specified	University or Academic Institution	Government	Other	Medicine or Public Health	Ecology or Environmental Sciences	Other	Government	Non-Government	Unsponsored	Not Reported	
Asia (cont.)	Kan, 1986	X								X	X			X						X	
	Kim, 1986		X					X				X		X						X	
	Hashimoto, 1989	X					X				X				X		X				
	MacLean, 1989				X					X			X		X					X	
	Swaddiwudhipong, 1989	X								X	X			X						X	
	Cheng, 1991	X								X	X			X						X	
	Viviani, 1992				X					X	X			X						X	
	Negoro, 1993	X								X	X	X		X						X	
	Hartigan-Go, 1994		X					X				X		X					X		
	Todd, 1994				X					X		X		X						X	
	Corrales, 1995				X					X	X		X		X		X	X			
	Hwang, 1995		X							X	X				X					X	
	Akaeda, 1998		X					X			X	X			X					X	X

Continent	First Author, Year	Study Design					Case Definition				Author Institutional Affiliation			Author Departmental Affiliation			Funding/ Sponsorship Source				Generalizability
		Ecologic or Time-series or Surveillance	Case series	Case Report	Literature Review	Other	Definite	Probable	Possible	Not Specified	University or Academic Institution	Government	Other	Medicine or Public Health	Ecology or Environmental Sciences	Other	Government	Non-Government	Unsponsored	Not Reported	
Asia (cont.)	Trevino, 1998				X					X			X	X						X	
	Bankoff, 1999				X					X	X					X				X	
	Morris Jr., 1999				X					X	X			X						X	
	Murakami, 2000	X					X					X		X		X				X	
	Azanza, 2001		X					X					X		X					X	
	Bajarias, 2002				X					X	X	X			X					X	
	Holmes, 2002				X					X	X				X		X				
	Batoréu, 2005		X					X			X			X						X	
	Azanza, 2006	X								X	X			X						X	X
	Chung, 2006					X		X			X	X		X						X	
	Jen, 2008		X					X			X	X		X	X					X	
	James, 2010				X					X	X			X	X		X				
	Toda, 2012		X				X				X			X						X	
	Ching, 2015		X					X				X		X						X	X

Continent	First Author, Year	Study Design					Case Definition				Author Institutional Affiliation			Author Departmental Affiliation			Funding/ Sponsorship Source				Generalizability
		Ecologic or Time-series or Surveillance	Case series	Case Report	Literature Review	Other	Definite	Probable	Possible	Not Specified	University or Academic Institution	Government	Other	Medicine or Public Health	Ecology or Environmental Sciences	Other	Government	Non-Government	Unsponsored	Not Reported	
Asia (cont.)	Suleiman, 2017		X							X	X	X		X						X	
	Arnich, 2018				X					X		X		X			X				
	Azzeri, 2020				X			X			X			X	X	X	X				
	Velayudhan, 2021		X							X	X			X						X	
	Chen, 2023		X				X		X			X		X			X				
	Yu, 2023	X								X	X	X			X		X				
	Zheng, 2023		X				X				X	X		X			X				
Europe	McFarren, 1960				X					X		X		X		X				X	
	McCollum, 1968	X								X	X			X	X					X	X
	Ayres, 1975	X					X					X			X		X				
	Blanc, 1977				X					X			X	X						X	
	Zwahlen, 1977		X				X					X		X						X	
	Blanc, 1978		X							X	X	X	X	X			X				
	Caroli, 1978		X				X					X		X						X	
	Imbert, 1979	X								X	X	X				X				X	

Continent	First Author, Year	Study Design					Case Definition				Author Institutional Affiliation			Author Departmental Affiliation			Funding/ Sponsorship Source				Generalizability
		Ecologic or Time-series or Surveillance	Case series	Case Report	Literature Review	Other	Definite	Probable	Possible	Not Specified	University or Academic Institution	Government	Other	Medicine or Public Health	Ecology or Environmental Sciences	Other	Government	Non-Government	Unsponsored	Not Reported	
Europe (cont.)	Gulbrandsen, 1981		X					X			X			X						X	
	Tangen, 1983	X					X					X			X		X				
	Hasselgård, 1984	X							X			X		X						X	
	Langeland, 1984		X					X			X			X						X	X
	Sanders, 1987				X					X	X			X						X	
	Mills, 1988				X					X			X			X				X	
	None Listed, 1988		X				X					X		X			X				
	The PHLS Communicable Disease Surveillance Centre, 1990	X					X					X		X			X				
	Scoging, 1991				X					X		X		X						X	
	Viviani, 1992				X					X	X			X						X	
	Ledoux, 1994				X					X		X		X		X				X	X
	Martin, 1996				X					X	X			X						X	

Continent	First Author, Year	Study Design					Case Definition				Author Institutional Affiliation			Author Departmental Affiliation			Funding/ Sponsorship Source				Generalizability
		Ecologic or Time-series or Surveillance	Case series	Case Report	Literature Review	Other	Definite	Probable	Possible	Not Specified	University or Academic Institution	Government	Other	Medicine or Public Health	Ecology or Environmental Sciences	Other	Government	Non-Government	Unsponsored	Not Reported	
Europe (cont.)	de Carvalho, 1998	X								X	X	X		X						X	
	Scoging, 1998				X					X		X		X						X	
	Krys, 2002				X					X		X		X						X	
	Batoréu, 2005		X					X			X			X						X	
	Mira Gutiérrez, 2005				X					X	X			X						X	
	Rapala, 2005	X						X			X	X		X	X				X		
	James, 2010				X					X	X			X	X		X				
	Hinder, 2011		X				X				X	X		X	X					X	
	Carvalho, 2019		X				X						X	X	X					X	
	Arnich, 2018				X					X		X		X			X				
	Vale, 2020		X				X					X			X		X				
	Karlson, 2021				X		X					X		X	X				X		
	Sinno-Tellier, 2022	X								X		X		X	X					X	
	Rodríguez, 2024	X								X	X	X			X		X				

Continent	First Author, Year	Study Design					Case Definition				Author Institutional Affiliation			Author Departmental Affiliation			Funding/ Sponsorship Source				Generalizability
		Ecologic or Time-series or Surveillance	Case series	Case Report	Literature Review	Other	Definite	Probable	Possible	Not Specified	University or Academic Institution	Government	Other	Medicine or Public Health	Ecology or Environmental Sciences	Other	Government	Non-Government	Unsponsored	Not Reported	
North America	Scobey, 1947				X			X					X			X				X	
	Meyers, 1955			X					X			X		X						X	
	Tennant, 1955		X						X			X		X	X					X	
	Bond, 1958		X				X					X			X					X	
	McFarren, 1960				X					X		X		X		X				X	
	Meinke 3rd, 1973			X					X		X		X	X						X	
	Fortuine, 1975		X						X				X	X						X	
	Cladouhos, 1977	X						X				X		X						X	X
	Craun, 1977		X				X					X		X	X					X	
	Hughes, 1977	X					X					X		X						X	
	Morse, 1977				X					X	X					X				X	
	Todd, 1977		X				X					X		X						X	
	Acres, 1978		X				X				X	X		X			X	X			X
	Imbert, 1979	X								X	X	X				X				X	

Continent	First Author, Year	Study Design					Case Definition				Author Institutional Affiliation			Author Departmental Affiliation			Funding/ Sponsorship Source				Generalizability
		Ecologic or Time-series or Surveillance	Case series	Case Report	Literature Review	Other	Definite	Probable	Possible	Not Specified	University or Academic Institution	Government	Other	Medicine or Public Health	Ecology or Environmental Sciences	Other	Government	Non-Government	Unsponsored	Not Reported	
North America (cont.)	Bryan, 1980	X								X		X		X						X	
	Grimard, 1981	X								X	X	X		X			X				
	Todd, 1982	X								X		X		X						X	
	de la Garza Aguilar, 1983		X				X					X		X						X	
	None Listed, 1983	X								X		X		X						X	
	Conte, 1984	X								X	X	X			X					X	X
	Todd, 1985a	X								X		X		X						X	
	Todd, 1985b	X								X		X		X						X	
	Mee, 1986		X				X					X		X	X					X	
	Sanders, 1987				X					X	X			X						X	
	Todd, 1987	X								X		X		X						X	
	Mills, 1988				X					X			X			X				X	
	None Listed, 1988		X				X					X		X			X				
	Todd, 1988	X								X		X		X						X	

Continent	First Author, Year	Study Design					Case Definition				Author Institutional Affiliation			Author Departmental Affiliation			Funding/ Sponsorship Source				Generalizability
		Ecologic or Time-series or Surveillance	Case series	Case Report	Literature Review	Other	Definite	Probable	Possible	Not Specified	University or Academic Institution	Government	Other	Medicine or Public Health	Ecology or Environmental Sciences	Other	Government	Non-Government	Unsponsored	Not Reported	
North America (cont.)	Todd, 1989	X								X		X		X						X	
	Long, 1990			X			X				X			X						X	
	Mata, 1990		X				X					X		X	X					X	X
	None Listed, 1990		X				X				X	X		X			X				
	Rodrigue, 1990	X						X			X	X		X						X	
	Ahmed, 1991				X					X			X	X			X				
	None Listed, 1991	X							X		X			X						X	
	Saldate Castañeda, 1991		X					X				X		X						X	
	United States Centers for Disease Control and Prevention, 1991	X					X					X		X						X	
	Ahmed, 1992	X								X		X		X						X	
	Viviani, 1992				X					X	X			X						X	
	Moss, 1993				X					X	X					X				X	
	Saavedra-Deigado, 1993				X					X		X		X						X	

Continent	First Author, Year	Study Design					Case Definition				Author Institutional Affiliation			Author Departmental Affiliation			Funding/ Sponsorship Source				Generalizability
		Ecologic or Time-series or Surveillance	Case series	Case Report	Literature Review	Other	Definite	Probable	Possible	Not Specified	University or Academic Institution	Government	Other	Medicine or Public Health	Ecology or Environmental Sciences	Other	Government	Non-Government	Unsponsored	Not Reported	
North America (cont.)	Todd, 1993	X								X	X			X			X				
	Cortés-Altamirano, 1993				X					X	X				X		X				
	Gessner, 1995		X				X				X	X		X			X				
	Bean, 1996	X								X		X		X						X	
	Gessner, 1996	X								X	X			X						X	X
	Bean, 1997		X					X				X		X						X	
	Gessner, 1997a		X				X				X	X		X	X					X	
	Gessner, 1997b			X			X					X		X	X					X	
	Todd, 1997		X				X				X	X		X				X			
	Sierra-Beltrán, 1998				X					X		X			X					X	
	Trevino, 1998				X					X			X			X				X	
	Bankoff, 1999				X					X	X			X						X	
	Morris Jr., 1999				X					X	X			X						X	
	United States Centers for Disease Control and Prevention, 2002a		X				X					X		X						X	X

Continent	First Author, Year	Study Design					Case Definition				Author Institutional Affiliation			Author Departmental Affiliation			Funding/ Sponsorship Source				Generalizability
		Ecologic or Time-series or Surveillance	Case series	Case Report	Literature Review	Other	Definite	Probable	Possible	Not Specified	University or Academic Institution	Government	Other	Medicine or Public Health	Ecology or Environmental Sciences	Other	Government	Non-Government	Unsponsored	Not Reported	
North America (cont.)	United States Centers for Disease Control and Prevention, 2002b	X						X				X		X						X	X
	Balmer-Hanchey, 2003				X					X	X			X	X		X				
	Barbier, 2003				X					X	X			X			X	X			
	Batoréu, 2005		X					X			X			X						X	
	Sobel, 2005				X					X		X		X						X	
	Fortuine, 2007		X					X				X		X						X	X
	Wang, 2008				X					X	X	X			X		X				
	Barraza, 2009		X				X					X			X					X	
	James, 2010				X					X	X			X	X		X				
	Bienfang, 2011				X			X			X	X	X	X	X	X	X				
	McLaughlin, 2011		X				X				X	X		X						X	
	Gould, 2013	X					X					X		X						X	

Continent	First Author, Year	Study Design					Case Definition				Author Institutional Affiliation			Author Departmental Affiliation			Funding/ Sponsorship Source				Generalizability
		Ecologic or Time-series or Surveillance	Case series	Case Report	Literature Review	Other	Definite	Probable	Possible	Not Specified	University or Academic Institution	Government	Other	Medicine or Public Health	Ecology or Environmental Sciences	Other	Government	Non-Government	Unsponsored	Not Reported	
North America (cont.)	DeGrasse, 2014		X				X					X		X	X		X				
	Hurley, 2014		X					X				X		X						X	
	Trainer, 2014				X			X			X	X	X		X					X	
	Callejas, 2015	X						X				X		X			X				X
	Clemence, 2015				X					X	X		X	X						X	
	Knaack, 2016		X				X				X	X		X	X					X	
	Arnich, 2018				X					X		X		X			X				
	Coleman, 2018			X			X					X		X	X		X				
	Anderson, 2021	X								X		X			X		X				X
	McIntyre, 2021		X				X				X			X	X					X	X
	Sunesen, 2021	X								X	X	X		X	X					X	
	Temple, 2022			X			X				X			X						X	
	Reséndiz-Colorado, 2023	X								X	X				X		X				
Oceania	McFarren, 1960				X					X		X		X		X				X	
	Rhodes, 1975		X				X				X	X		X			X				
	Eason, 1987		X				X				X			X						X	X

Continent	First Author, Year	Study Design					Case Definition				Author Institutional Affiliation			Author Departmental Affiliation			Funding/ Sponsorship Source				Generalizability
		Ecologic or Time-series or Surveillance	Case series	Case Report	Literature Review	Other	Definite	Probable	Possible	Not Specified	University or Academic Institution	Government	Other	Medicine or Public Health	Ecology or Environmental Sciences	Other	Government	Non-Government	Unsponsored	Not Reported	
Oceania (cont.)	MacLean, 1989				X					X			X		X					X	
	Bankoff, 1999				X					X	X			X						X	
	Lehane, 2001				X				X			X				X				X	
	Turnbull, 2013			X				X				X		X	X				X		
	Arnich, 2018				X					X		X		X			X				
	Edwards, 2018		X				X				X	X		X	X		X				X
South America	Vecchio, 1986	X								X	X	X		X						X	
	Montebruno, 1993a		X					X					X	X						X	
	Montebruno, 1993b	X								X		X		X						X	
	García, 2004		X				X				X	X		X						X	
	La Barbera-Sánchez, 2004					X				X	X	X			X	X	X				

Continent	First Author, Year	Study Design					Case Definition				Author Institutional Affiliation			Author Departmental Affiliation			Funding/ Sponsorship Source				Generalizability
		Ecologic or Time-series or Surveillance	Case series	Case Report	Literature Review	Other	Definite	Probable	Possible	Not Specified	University or Academic Institution	Government	Other	Medicine or Public Health	Ecology or Environmental Sciences	Other	Government	Non-Government	Unsponsored	Not Reported	
South America (cont.)	Batoréu, 2005		X					X			X			X						X	
	García, 2005		X				X					X		X		X				X	
	Hernández, 2005	X								X	X	X		X						X	
	James, 2010				X					X	X			X	X		X				
	Arnich, 2018				X					X		X		X			X				
	Sunesen, 2021	X								X	X	X		X	X					X	
	Barría, 2022	X								X	X	X		X	X		X			X	
	Mafra Jr., 2023	X								X	X	X			X		X				
Global	Lehane, 2001				X				X			X				X				X	
	Sinno-Tellier, 2023	X								X	X	X		X						X	

Text S1. Data Extraction Form. This form was used to collect information from all articles that fit our search criteria. The form was categorized to collect data on the study identification, outbreak details, and case demo

A. STUDY IDENTIFICATION DATA

1. **Study ID Number (i.e., Covidence assigned number)**

2. **Last Name of First Author**

3. **Year of Publication**

4. **Journal Name**

5. **Author (include all authors on publication) Institutional Affiliation (Check all that apply)**	**Yes/No**
University/Academic Institution	
Government	
Other	

6. **Author (include all authors on publication) Departmental Affiliation (Check all that apply)**	**Yes/No**
Medicine/Public Health	
Ecology/Environmental Science	
Other	

7. **Study Funding/Sponsorship (Check all that apply)**	**Yes/No**
Government (including grants from federal sources)	
Non-government (including grants from private sources)	
Unspecified	

8. **Language of Article**
   a.

9. **Number of outbreaks reported**

10. Outbreak Data Table	Outbreak 1	Outbreak 2	Outbreak 3	Outbreak 4	Outbreak 5	Outbreak 6	Outbreak 7	Outbreak 8	Outbreak 9	Outbreak 10	Outbreak 11	Outbreak 12	Outbreak 13	Outbreak 14	Outbreak 15
**Number of Cases**															
**Outbreak Start (Month/Year)**															
**Outbreak End (Month/Year)**															
**Duration of Symptoms**															
**Number of Cases Hospitalized**															
**Number requiring intensive care/intubation/ventilator**															
**Duration of Hospitalization**															
**Number Died**															
**Time of Death**															
**Number Female**															
**Number Male**															
**Country**															
**State/City**															

11. Study design	Select one
Ecologic/Time-series/Surveillance (Summary statistics over time and space)	
Case series (Presents data on n>1 case/person at an individual level)	
Case report (Presents data on n=1 case/person at an individual level)	
Other	

B. OUTBREAK DETAILS

12. Case Definition (if multiple apply, please choose the most confirmed case)	Select one
**Definite case** (Symptoms attributed to PSP after seafood eaten AND saxitoxin biomarker confirmed **-** human urine or in seafood eaten)	
**Probable case** (Symptoms attributed to PSP after seafood eaten but NO saxitoxin biomarker confirmation)	
Possible case (Symptoms noted as possibly consistent with PSP after seafood eaten and NO saxitoxin biomarker confirmation)	
**Possible non-seafood-related case** (Symptoms noted as possibly consistent with PSP without seafood exposure and NO saxitoxin biomarker confirmation)	
**Not specified** (e.g. surveillance reports with total PSP numbers in a decade etc.)	

13. Reported symptoms of cases (Check all that apply)	Yes/No
Tingling/paresthesias of lips/tongue	
Tingling/paresthesias of arms/hands/legs/toes	
Weakness/ataxia (loss of control/lack of coordination) of arms/legs	
Nausea/Vomiting	
Headache	
Dizziness	
Dysarthria (difficulty speaking)	
Dysphagia (difficulty swallowing)	
Difficulty breathing/respiratory failure	

13. Reported symptoms of cases (Check all that apply)	Yes/No
Paralysis	
Death	

14. Other symptoms (not listed in above choices)

15. Risk Factors/Exposures (What did the cases eat?) (Check all that apply)	Yes/No
Mollusks/Shellfish	
Fish	
Algae/Water	
Unspecified	

16. Specific food/Specific exposure source

17. What toxigenic algae was responsible for the poisoning (genus; e.g., Alexandrium)?

18. Exposure Location	Yes/No
Home (paper notes people live local to outbreak location)	
Vacation/Tourists (paper notes people were visiting outbreak location)	
Not reported	

19. Origin of Shellfish	Yes/No
Personal fishing/clamming/subsistence harvesting	
Bought at market/store	
Consumed in restaurant/catered party (unknown how restaurant/catering got shellfish)	
Not reported	

20. Time from Exposure to Symptom Onset (Check earliest time window that applies to reported cases)	Select one
<30 minutes	

20. Time from Exposure to Symptom Onset (Check earliest time window that applies to reported cases)	Select one
30 minutes to 1 hour	
1-4 hours	
>4 to 12 hours	
>12 hours	
Not reported	

C. DEMOGRPAHICS OF CASES

21. Ages of Cases Reported (Check all that apply)	Yes/No
0-9 years	
10-19 years	
20-29 years	
30-39 years	
40-49 years	
50-59 years	
60-69 years	
70-79 years	
80+ years	

22. Race/Ethnicity of Cases Reported	Count
**White**	
**Black**	
**Hispanic**	
**Native/Indigenous**	
**Asian**	
**Other**	

23. Generalizability (Target Population)

24. Do the authors define the target population for inference (yes/no)? (i.e., do the authors define the population to whom the results of the study apply?)

Figure S1. Clinical Characteristics. Geographic distribution of symptoms from articles reporting outbreaks on a single continent. Sinno-Tellier et al. 2023 (141) was excluded due to indiscriminate geography. Similarly, all articles reporting outbreaks from multiple continents (n=16) were excluded due to no symptom stratification by continent.

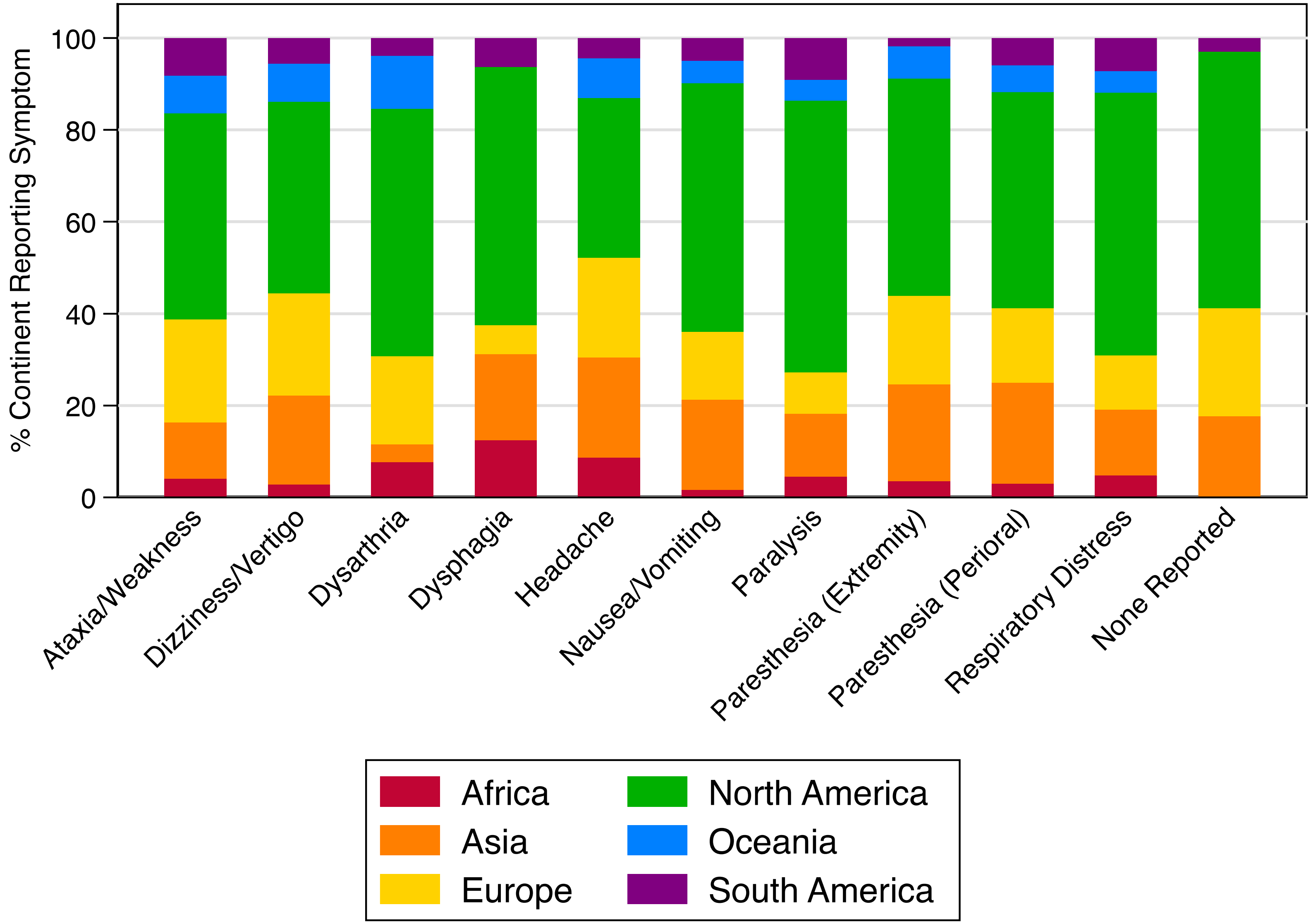

Supplement: Supplementary appendix [file NIHMS2112751-supplement-Supplementary_appendix.pdf]
